# Conservation of polypyrimidine tract binding proteins and their putative target RNAs in several storage root crops

**DOI:** 10.1186/s12864-018-4502-7

**Published:** 2018-02-07

**Authors:** Kirtikumar R. Kondhare, Amit Kumar, David J. Hannapel, Anjan K. Banerjee

**Affiliations:** 1 Biology Division, Indian Institute of Science Education and Research (IISER) Pune, Dr. Homi Bhabha Road, Pune, Maharashtra 411008 India; 2 Plant Biology Major, 253 Horticulture Hall, Iowa State University, Ames, IA 50011-1100 USA

**Keywords:** Phloem mobile, Signaling, Potato, BEL1-like, KNOX, Storage root crops

## Abstract

**Background:**

Polypyrimidine-tract binding proteins (PTBs) are ubiquitous RNA-binding proteins in plants and animals that play diverse role in RNA metabolic processes. PTB proteins bind to target RNAs through motifs rich in cytosine/uracil residues to fine-tune transcript metabolism. Among tuber and root crops, potato has been widely studied to understand the mobile signals that activate tuber development. Potato PTBs, designated as StPTB1 and StPTB6, function in a long-distance transport system by binding to specific mRNAs (*StBEL5* and *POTH1*) to stabilize them and facilitate their movement from leaf to stolon, the site of tuber induction, where they activate tuber and root growth. Storage tubers and root crops are important sustenance food crops grown throughout the world. Despite the availability of genome sequence for sweet potato, cassava, carrot and sugar beet, the molecular mechanism of root-derived storage organ development remains completely unexplored. Considering the pivotal role of PTBs and their target RNAs in potato storage organ development, we propose that a similar mechanism may be prevalent in storage root crops as well.

**Results:**

Through a bioinformatics survey utilizing available genome databases, we identify the orthologues of potato PTB proteins and two phloem-mobile RNAs, *StBEL5* and *POTH1,* in five storage root crops - sweet potato, cassava, carrot, radish and sugar beet. Like potato, PTB1/6 type proteins from these storage root crops contain four conserved RNA Recognition Motifs (characteristic of RNA-binding PTBs) in their protein sequences. Further, 3´ UTR (untranslated region) analysis of *BEL5* and *POTH1* orthologues revealed the presence of several cytosine/uracil motifs, similar to those present in potato *StBEL5* and *POTH1* RNAs. Using RT-qPCR assays, we verified the presence of these related transcripts in leaf and root tissues of these five storage root crops. Similar to potato, *BEL5-*, *PTB1/6-* and *POTH1*-like orthologue RNAs from the aforementioned storage root crops exhibited differential accumulation patterns in leaf and storage root tissues.

**Conclusions:**

Our results suggest that the PTB1/6-like orthologues and their putative targets, *BEL5-* and *POTH1*-*like* mRNAs, from storage root crops could interact physically, similar to that in potato, and potentially, could function as key molecular signals controlling storage organ development in root crops.

**Electronic supplementary material:**

The online version of this article (10.1186/s12864-018-4502-7) contains supplementary material, which is available to authorized users.

## Background

Polypyrimidine tract-binding proteins (PTBs) are ubiquitous RNA-binding proteins (RBPs) in eukaryotes [1, 2]. PTBs bind to select target RNAs to facilitate RNA metabolic and transport processes including mRNA polyadenylation [3], splicing repression in pre-mRNAs [4], RNA transport [5], mRNA stability/decay [6], and translational control [7]. The amino terminus of PTBs contain two distinct regions: a nuclear export signal and a nuclear localization signal [8]. Most PTB proteins are present in the nucleus, but in some systems, PTBs shuttle rapidly between the nucleus and cytoplasm [8]. PTBs contain four RNA-recognition motifs (RRMs), designated RRM1, RRM2, RRM3, and RRM4 [1]. These RRMs are approximately 90 amino acids in length and are connected by varying lengths of linker sequences. Each RRM is formed by four to five β-sheets and contain six to eight conserved amino acids, designated Ribonucleoprotein1 (RNP1) and RNP2, that interact with CU (cytosine/uracil) motifs, ranging from three to five nucleotides in length present in target RNAs [1, 2]. RRM1 and RRM2 function independently, whereas RRM3 and RRM4 work in a tandem complex that functions as an open-faced clamp on closely spaced polypyrimidine-tract motifs [1, 2]. Each RRM has a slightly different consensus RNA-binding sequence, but they all recognize short pyrimidine (CU) sequences [9]. The ability of these RRMs to bind different sequences on the same RNA molecule allows them to function as RNA remodelers. The interaction between RRMs and target RNAs brings separated pyrimidine tracts into close proximity making loops in the structure of target RNAs [1]. Although PTBs exhibit widespread function and versatility in eukaryotes, very little is known about their role in plant development.

A large number of RBPs with a wide range of functions has been catalogued in plants [10, 11]. PHLOEM PROTEIN16 (CmPP16) and the PTB protein, CmRBP50, were the first RBPs to be identified in the phloem sap of pumpkin [5, 12]. CmRBP50 functions as the core member in a phloem-mobile RNA/protein complex that consists of 16 proteins and six RNAs. Gel mobility-shift assays confirmed the binding of CmRBP50 to phloem mRNAs such as *CmGAI* and *CmPP16–1* and demonstrated this interaction was mediated by ‘CU’ sequences located within the 5´ and 3´ untranslated regions (UTRs) [5]. Of the three PTBs in Arabidopsis (AtPTB1, − 2 and − 3), AtPTB3 is most closely related to CmRBP50. Both these latter types contain four RRMs [5, 13], whereas AtPTB1 and − 2 contain only three. AtPTB1 and − 2 are mainly involved in alternative splicing (AS) [13]. No significant AS regulatory function, however, was observed for the distantly related AtPTB3 [13–15].

Six PTB family genes have been identified in potato, designated StPTB1, − 6, − 7, − 7.1, − 7.2 and − 7.3 [16]. Based on protein sequence, they may be grouped into two clades. The first clade has two members, StPTB1 and StPTB6, which have approximately 85% amino-acid sequence identity match to CmRBP50 and AtPTB3. It is postulated that this group functions as chaperones to full-length mRNAs that are transported through the sieve element system [5, 16]. The second StPTB clade, designated the PTB7 clade, has four members that share close identity with AtPTB1 and AtPTB2 [16]. All six of these PTBs lack a conserved RRM4 and are proposed to have a different function than the CmRBP50 and AtPTB3 types [17]. StPTB1 and StPTB6 bind to *StBEL5* RNA and provide stability to its transcript during its transport to stolon tips and roots [16]. StBEL5 is a transcription factor that plays a pivotal upstream role in tuber formation [18]. Binding and movement assays have shown that cytosine/uracil motifs predominately present in the 3´ UTRs of *StBEL5* appeared to be most critical in transporting its RNA from leaves into the stolon tips in potato [16, 19, 20]. Despite the importance and ubiquity of the PTBs, very little information is available on their biological function at the whole-plant level. Recent results, however, suggest that PTB proteins function in a range of processes involving alternative splicing and long-distance transport of select transcripts that impact development in plants [5, 13, 15, 16, 21]. We will now discuss in more detail the role of specific PTB proteins of potato that function as chaperones in the delivery of a key mobile signal that activates tuber formation.

BEL1- and KNOTTED1-type homeobox proteins are transcription factors from the three-amino-loop-extension (TALE) superfamily [22] that interact to mediate expression of select target genes. BEL-like genes are ubiquitous in plants and function in a wide range of developmental processes [18, 23–26]. StBEL5 and its KNOTTED1-type protein partner, POTH1, regulate tuberization by targeting genes that control growth processes of the tuber pathway [27]. The over-expression of POTH1 in transgenic lines produces earliness during in vitro tuberization [28], suggesting a positive role of POTH1 in tuber formation. The transcript of *POTH1* is also found to be phloem mobile and its UTRs bind to two RBPs, StPTB6 and an alba-domain type [29].

In potato, *StBEL5* mRNA functions as a phloem-mobile signal that activates tuberization [19]. This movement is enhanced under short-day conditions and is mediated by motifs present in the UTRs of *StBEL5* [19, 20, 30]. Potato PTBs in the same class as CmRBP50, designated StPTB1 and StPTB6, were confirmed to bind to *StBEL5* mRNA through CU motifs present in its 3´ UTR. This interaction enhances transcript stability, mediates long-distance transport from the source leaf, and promotes localization of *StBEL5* to stolon tips and roots [16]. StPTB1 and StPTB6 over-expression lines of potato exhibited increased tuber yields, whereas RNA suppression lines showed a significant reduction in tuber yields [16]. This positive effect of StPTB1 and StPTB6 on tuber formation was indirectly controlled by enhancing levels of *StBEL5* transcripts [16]. Similar to *StBEL5* RNA, a recent study has demonstrated that the transcripts of two genes closely related to *StBEL5*, *StBEL11* and *− 29*, are also phloem mobile, but they act as repressors of tuberization [31]. Despite their antagonistic relationship, the movement of all three of these RNAs is enhanced by short days and each of the three contain an abundant number of CU motifs in their 3´ UTRs. *StBEL5* contains sixteen, whereas *StBEL11* and *− 29* contain seven and eleven CU motifs, respectively [32]. Consistent with this latter observation, the activity of StPTB1 and StPTB6 is strongly correlated with movement of *StBEL11* and *− 29* into both stolons and roots [31]. Overall, these results suggest that *StBEL5*, *− 11*, and *− 29* function in concert to balance growth during tuber and root formation and that their long-distance phloem transport may be mediated by the same molecular process [31, 32]. Because this StBEL/StPTB signal complex regulates underground organ development in potato [30, 31, 33, 34], it is conceivable that similar genetic pathways are conserved in regulating the formation of other storage organs.

Storage organs in plants may develop as tubers (yams, potato) or roots (cassava, sweet potato, sugar beet, radish) and serve as an essential food in both tropical and temperate areas of the world. Four of the top ten world food crops (https://www.nationalgeographic.org/maps/wbt-staple-food-crops-world/) are storage roots or tubers. They produce very high caloric yields per area of cultivation, represent a healthy nutrient source, are very easy to grow, and generate significant income for local farmers. They are rich in beta-carotenes, calcium, vitamin A, B, and C, iron, iodine, fructan, storage proteins and starch. In addition, many tuber and root crops exhibit antioxidative, hypoglycemic, hypocholesterolemic, antimicrobial, and immunomodulatory properties [35]. They also have diverse numerous applications in the paper, fabric and starch adhesives industries [36]. Several root crops exhibit an immense potential as functional foods and nutraceutical ingredients to be explored in disease risk reduction and wellness [35]. Despite the enormous importance of tuber and root crops, except for potato [33], our understanding of the signaling mechanisms that regulate underground storage organ development is lacking. As complete genomic sequences become available, opportunities for establishing conserved growth processes in diverse food crop groups based on genetic and bioinformatic approaches become readily apparent. In potato, several studies have shown that *StBEL* mRNAs and PTB proteins play a significant signaling role in tuber formation [16, 19, 20, 29, 31]. There has been no attempt, however, to address the question of whether or not the StBEL and PTB components are conserved in the genome of any other storage organ food crops. In this context, potato tuber formation and its signal components may be utilized as a model to explore this question in more detail. In this study, our goal was to establish the conservation of BEL5, POTH1, and PTB orthologues in the genomes of five storage root crops. If they do exist, it would be compelling to explore their potential role (similar to potato) as signals in the regulation of storage organ development.

## Methods

### Identifying orthologues of StBEL5, POTH1, StPTB1 and − 6 in storage root crops

RNA and protein sequences of *POTH1*, *StBEL5*, *StPTB1* and *− 6* orthologue genes in storage root crops, carrot (*Daucus carota*), radish (*Raphanus sativus*) and sugar beet (*Beta vulgaris*), were obtained from NCBI by protein BLAST suite using respective potato protein sequences as queries (https://blast.ncbi.nlm.nih.gov/Blast.cgi?PROGRAM=blastp&PAGE_TYPE=BlastSearch&LINK_LOC=blasthome). RNA and protein sequences for cassava (*Manihot esculenta*) were retrieved from the Plant Genomics Resource database with Phytozome version 12.0 (https://phytozome.jgi.doe.gov/pz/portal.html) and for sweet potato (*Ipomoea trifida*) from the Sweet potato Genomics Resource database (http://sweetpotato.plantbiology.msu.edu/blast.shtml) with default filters and the expected threshold value of 1e^− 10^. All accession numbers are included in appropriate Figs. and Tables. Note: Genome sequencing and annotation has been difficult in sweet potato (*Ipomoea batatas* (L.) Lam) because of its hexaploid genome structure [37]. In this report, *I. trifida* is used as the reference genome. It is a diploid species and its genome has been sequenced and annotated. It is the closest wild species to sweet potato (Additional file 1: Figure S1**),** and its most likely ancestor [38, 39].

### Sequence alignment analysis for StPTB1 and − 6 orthologues in storage root crops

**Fig. 1:**
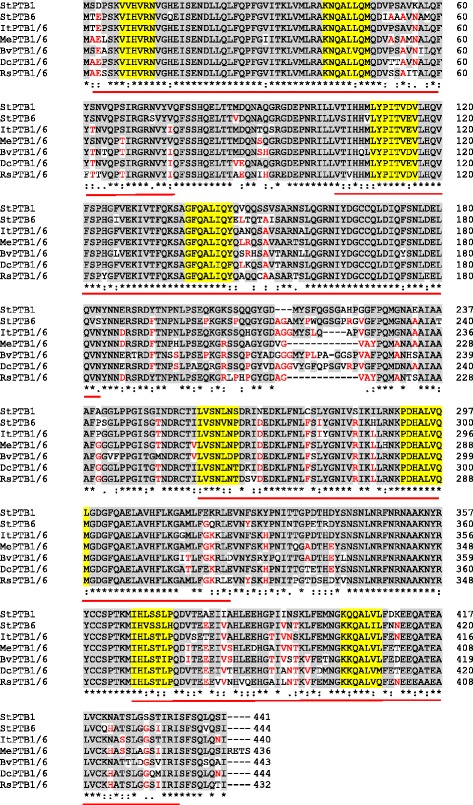
Amino-acid sequence alignment of StPTB1/6-like orthologues in select storage root crops*.* Gray boxed letters represent the residues in PTB1/6-like orthologues of storage root crops identical to StPTB1 and StPTB6, letters highlighted in red represent the residues identical in at least two PTB1/6-like orthologues, whereas residues not highlighted represent non-conserved residues among these PTB1/6-like orthologues. Four RNA recognition motifs (RRMs) are underlined in red. Potential canonical RNPs in each RRM are highlighted in yellow. Clustal consensus sequences are represented by asterisks below the alignment. The amino-acid sequences of PTB1/6-like orthologues in storage root crops are aligned to StPTB1 and StPTB6 amino-acid sequences in potato as a reference. CmRBP50 RRM and RNP sequences were used for identifying potential RRM and RNPs in these StPTB1/6-like orthologues [5]. Among the different PTB1/6-like variants identified in each storage root crop (Table 1), one protein per crop with the best coverage and identity were considered for the sequence alignment shown here. These accessions for protein sequences were: ItPTB1/6-like (itf09g10450.t1), MePTB1/6-like (Manes.18G093400.1), DcPTB1/6-like (XP_017247842.1), RsPTB1/6-like (XP_018451916.1) and BvPTB1/6-like (XP_010681101.1). PTB, polypyrimidine tract-binding; St, *Solanum tuberosum*; It, *Ipomoea trifida*; Me, *Manihot esculenta*; Bv, *Beta vulgaris*; Dc, *Daucus carota*; Rs, *Raphanus sativus*

Protein sequences of PTB1/6-like orthologues from the storage root crops, sweet potato, cassava, carrot, radish and sugar beet were aligned to potato StPTB1 and StPTB6 amino-acid sequences as reference. Multiple sequence alignments were performed using Clustal Omega2 software [40] with default parameters (https://www.ebi.ac.uk/Tools/msa/clustalo) and the alignment files were manually edited. Four RNA recognition motifs (RRMs) and potential canonical RNPs in each RRM are shown in Fig. 1. RRM and RNP motifs shown for StPTB1 and StPTB6 were derived from CmRBP50 sequence [5] and were used as references in identifying putative RRM and RNPs in the PTB1/6-like orthologues from the five storage root crops.

### Sample harvest and RNA detection assays

Leaf and storage root samples of sweet potato, cassava, carrot, radish and sugar beet were harvested from the agricultural farm near the Pune institute. Species authentication was performed using trnS intergenic spacer sequence analysis (Additional file 2: Table S1). Leaves and roots of 2–3 plants each were pooled in triplicate for tissue grinding in liquid nitrogen. Total RNA from leaf and root tissues was isolated using RNAiso Plus (Takara-Clontech) with three biological replicates from the aforementioned storage root crops, except cassava. For cassava, total RNA was isolated by RNasey Plant Mini kit I (Qiagen). Potato (*S. tuberosum* ssp. *andigena*) plants were grown for three months under long-day conditions in a growth chamber (Percival Pvt. Ltd.). Potato leaf and root tissues were harvested and ground in liquid nitrogen. Two micrograms of RNA (DNase treated with RQ1 RNase-Free DNase; Cat. # M6101; Promega) were reverse-transcribed using oligo(dT) primer and SuperScript-IV reverse transcriptase (SS-IV RT; Invitrogen). All transcripts were detected by RT-PCR assays using undiluted cDNA and gene-specific primers (Additional file 3: Table S2). Reaction conditions were 95 °C for 3 min, followed by 35 cycles of 95 °C for 30 s, gene-specific annealing temperature for 30 s and 74 °C for 30 s, with final extension at 74 °C for 5 min. Amplified PCR products were purified using PCR Clean-up kit (Takara-Clonetech) and sequence verified.

### Real-time qPCR analysis

All RT-qPCR reactions were performed on a CFX96 Real-Time System (BIO-RAD) with gene-specific primers (Additional file 3: Table S2) and using the cDNAs synthesized above. For *StBEL5-*, *StPTB1/6-* and *POTH1-*like orthologues in the storage root crops, qPCR reactions were carried out with three biological replicates and three technical replicates. The reactions were carried out using TAKARA SYBR® green master mix (Takara-Clontech) and incubated at 95 °C for 3 min, followed by 40 cycles at 95 °C for 5 s, gene-specific annealing temperature for 15 s (Additional file 3: Table S2) and extension at 72 °C for 20 s. *GAPDH* was used for normalization for all the reactions (Fig. 5). PCR specificity was checked by melting curve analysis, and data were analysed using the 2^–ΔΔCt^ method [41]. Statistical analysis was carried out using a Student’s *t*-test with *p* ≤ 0.05.

### Phylogenetic analysis

The deduced amino-acid sequences for thirteen PTB1/6-like proteins from nine species including Arabidopsis, tobacco, tomato, potato, sweet potato, cassava, carrot, radish and sugar beet were obtained from NCBI and used to build a phylogenetic tree for CmRBP50-like PTBs. Amino-acid sequence alignments, phylogenetic analysis and graphical representation of the phylogenetic tree were performed using T-COFFEE with TreeDyn (v198.3) (http://phylogeny.lirmm.fr/phylo_cgi/simple_phylogeny.cgi) [42]. In the phylogenetic tree, the branch length is proportional to the number of substitutions per site and the tree was rerooted using midpoint rooting in TreeDyn. Amino-acid sequences of AtPTB2 (a distant homolog of Arabidopsis AtPTB3), HnRNPI (a human PTB protein) and StBMI1 (a potato non-PTB related protein) were included for rooting the phylogenetic trees. Conserved RRM (RNA recognition motif) domains characteristic of PTB proteins were also identified using BLAST for all PTB1/6-like proteins from the five storage root crops. Similarly, phylogenetic trees were built for POTH1- and BEL5-like orthologues in these five storage root crops.

## Results

### Identification of BEL5-, POTH1- and PTB1/6-like genes in storage root crops

**Fig. 2:**
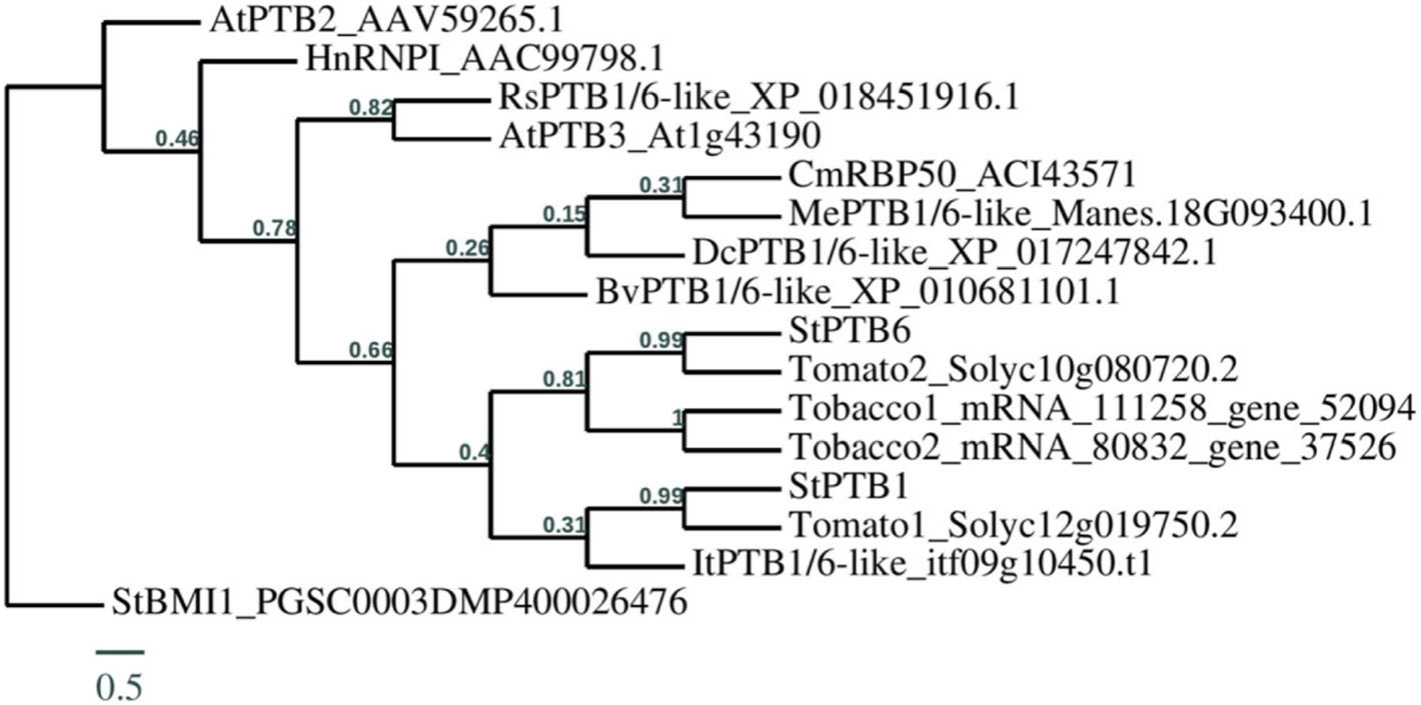
Phylogenetic relationship of RBP50-like PTBs from the Solanaceae family and PTB1/6-like proteins from five storage root crops (sweet potato, cassava, carrot, radish and sugar beet) selected from Table 1. For comparison, the deduced amino-acid sequences for thirteen PTB1/6-like proteins from nine species were analyzed. AtPTB2 (a distant homolog of Arabidopsis AtPTB3), HnRNPI (human PTB) and StBMI1 (a potato non-PTB related protein) amino-acid sequences are included as controls. Conserved RRM (RNA recognition motif) domains characteristic of PTB proteins were also identified using BLAST for all PTB1/6-like proteins from these storage root crops. Amino-acid sequence alignments and phylogenetic analysis were performed using T-COFFEE (hRp://www.ch.embnet.org/soaware/TCoffee.html) and graphical representation of the phylogenetic tree was performed with TreeDyn (v198.3) [42]. Accessions for protein sequences used are written after protein names in the phylogenetic tree. In the phylogenetic tree, the branch length is proportional to the number of substitutions per site and the tree is rerooted using midpoint rooting in TreeDyn. Bv, *Beta vulgaris*; Cm, *Cucurbita maxima*; Dc, *Daucus carota*; It, *Ipomoea trifida*; Me, *Manihot esculenta*; Rs, *Raphanus sativus*; St, *Solanum tuberosum*; PTB, polypyrimidine tract-binding

In order to characterize PTBs and two target RNAs in storage root crops, we sought putative orthologues through a BLAST search. Our data mining revealed that orthologues of both target RNAs and StPTB1/6-like proteins are present in the genomes of the root crops examined here. Using StPTB1 as the search sequence, several PTB proteins were identified (Table 1). Most contain only three RRMs and would be grouped with the StPTB7 types, assumedly involved in alternative splicing and other cell-autonomous processes related to RNA metabolism. Those proteins with four RRMs, two for cassava and sugar beet and only one for the other three, would be grouped into the StPTB1/6-CmRBP50 family of PTBs [16]. This latter group had a percent sequence identity match to StPTB1 ranging from 80 (sugar beet) to 88% (sweet potato). Multiple sequence alignment of StPTB1 and StPTB6 with select orthologues from these storage root crops revealed a high overall sequence match and a high level of concordance in the conserved sequence of the RRMs and RNPs (Fig. 1). Like CmRBP50, StPTB1 and StPTB6, the orthologues in these storage root crops contain two putative RNPs in each RRM (Fig. 1, yellow highlight). Alignments for all the PTB orthologues shown in Table 1 with StPTB1 and StPTB6 are shown in Additional file 4: Figure S2. Phylogenetic analyses of these PTBs shows their similarity and their relationship to other PTBs from a variety of plant species (Fig. 2). Potato, tomato, and tobacco each have two CmRBP50-type proteins, whereas Arabidopsis has only one, AtPTB3. As expected, the sweet potato orthologue (itf09g10450.t1), clusters within the StPTB1/6 clade, whereas the cassava orthologue (Manes.18G093400.1) and one sugar beet and one carrot type (XP_010681101.1 and XP_017247842.1) cluster more closely with CmRBP50 (Fig. 2).

**Table 1:** Potato PTB orthologues in five storage root crops

Crops	StPTB1/6 types (StPTB1-PGSC0003DMG400018824) (StPTB6-PGSC0003DMG400023660)	Coverage (%)	Identity (%)	No. of conserved RRMs	No. of PTB1/6 types	No. of PTB7 types
**Cassava** (*Manihot esculants*)	**Manes.18G093400.1** Manes.02G181600.1 Manes.14G018200.1 Manes.05G170900.1	99 99 94 43	84 83 42 28	4 4 3 3	2	2
**Sugar beet** (*Beta vulgaris*)	**XP_010681101.**1 XP_010680298.1 XP_010693257.1 XP_010671511.1 KMT16075.1 XP_010693256.1	99 99 97 61 61 97	80 72 32 42 42 31	4 4 3 3 3 3	2	4
**Raddish** (*Raphanus sativus*)	**XP_018451916.1** XP_018441449.1 XP_018441450.1 XP_018472559.1 XP_018488837.1 XP_018488838.1 XP_018488836.1 XP_018463656.1	99 98 90 64 93 89 93 93	80 28 32 42 31 32 29 31	4 3 3 3 3 3 3 3	1	7
**Carrot** (*Daucus carota*)	**XP_017247842.1** XP_017241710.1 XP_017242965.1 XP_017246841.1 KZM98104.1 KZN02333.1	99 61 91 91 91 60	85 43 30 30 30 43	4 3 3 3 3 3	1	5
**Sweet potato** (*Ipomoea trifida*)	**itf09g10450.t1** itf05g19430.t1 itf05g16040.t1 itf12g25960.t1 itf05g19430.t2	100 91 76 68 91	88 31 41 41 30	4 3 3 3 3	1	4

Orthologues of the potato PTB1/6 proteins in storage root crops. Among the PTB orthologues in each storage root crop, proteins with the best coverage and identity (highlighted in bold) were used in the multiple sequence alignment (Fig. 1) and phylogenetic analysis (Fig. 2). Sequence of StPTB1 was used for the query in this analysis. The same results were obtained using StPTB6 as a query because StPTB1 and StPTB6 amino-acid sequences have a close similarity. PTB orthologues with potential four RRMs are considered as PTB1/6-types, whereas those with three RRMs as PTB7 types

**Fig. 3:**
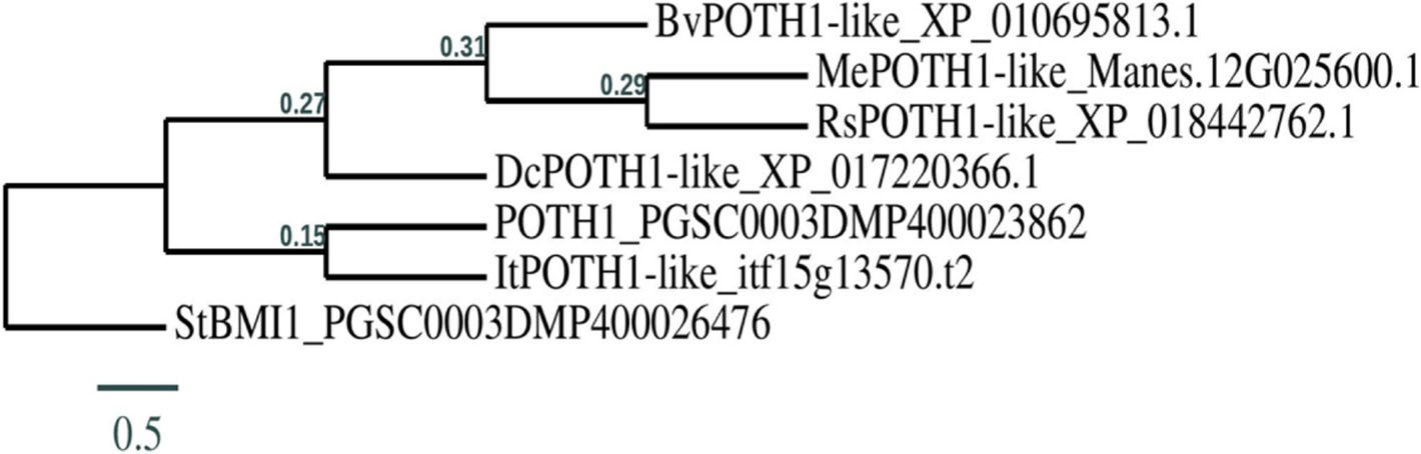
Phylogenetic relationship of POTH1-like proteins from five storage root crops (sweet potato, cassava, carrot, radish and sugar beet). For comparison, the deduced amino-acid sequences for POTH1-like proteins from five storage root crops plus POTH1 of potato were analyzed. StBMI1 (a potato non-POTH1 related protein) amino-acid sequences are included as controls. Amino-acid sequence alignments and phylogenetic analysis were performed using T-COFFEE (hRp://www.ch.embnet.org/ soaware/TCoffee.html) and graphical representation of the phylogenetic tree was performed with TreeDyn (v198.3) [42]. Accessions for protein sequences used are written after protein names in the phylogenetic tree. In the phylogenetic tree, the branch length is proportional to the number of substitutions per site and the tree is rerooted using midpoint rooting in TreeDyn. Bv, *Beta vulgaris*; Dc, *Daucus carota*; It, *Ipomoea trifida*; Me, *Manihot esculenta*; Rs, *Raphanus sativus*; St, *Solanum tuberosum*

**Fig. 4:**
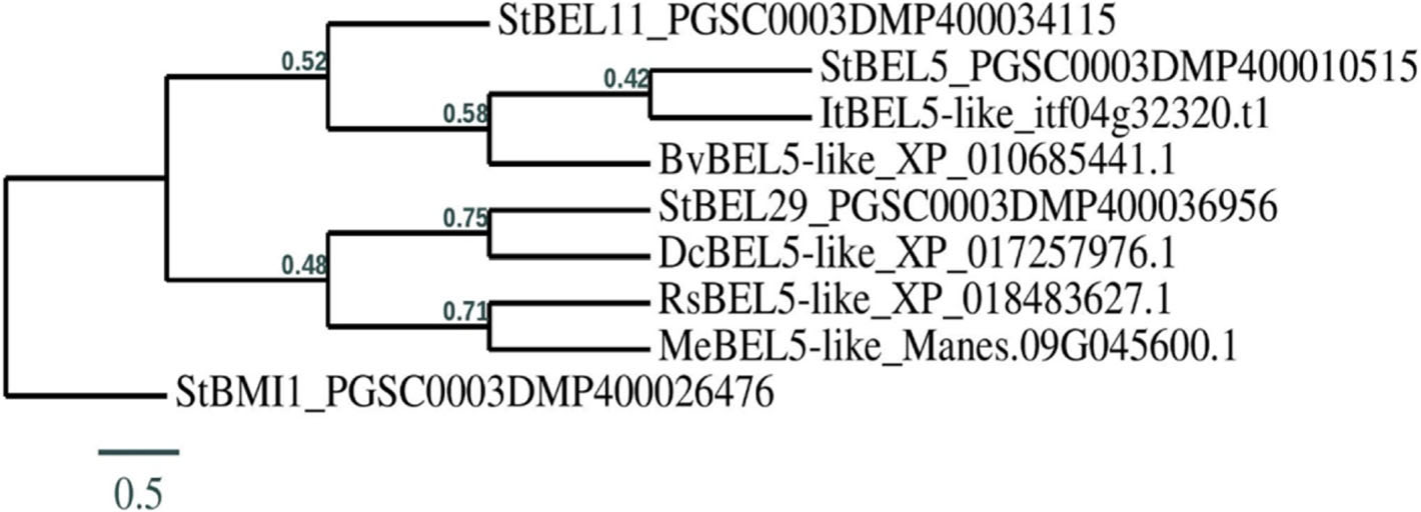
Phylogenetic relationship of BEL5-like proteins from several storage root crops (sweet potato, cassava, carrot, radish and sugar beet). For comparison, the deduced amino-acid sequences for BEL5-like proteins from the five storage root crops plus three from potato (BEL5, − 11, and − 29) were analyzed. StBMI1 (a potato non-BEL5 related protein) amino-acid sequences are included as controls. Amino-acid sequence alignments and phylogenetic analysis were performed using T-COFFEE (hRp://www.ch.embnet.org/soaware/TCoffee.html) and graphical representation of the phylogenetic tree was performed with TreeDyn (v198.3) [42]. Accessions for protein sequences used are written after protein names in the phylogenetic tree. In the phylogenetic tree, the branch length is proportional to the number of substitutions per site and the tree is rerooted using midpoint rooting in TreeDyn. Bv, *Beta vulgaris*; Dc, *Daucus carota*; It, *Ipomoea trifida*; Me, *Manihot esculenta*; Rs, *Raphanus sativus*; St, *Solanum tuberosum*

Because of StBEL5’s role as a mobile signal for tuber development [19] and the involvement of KNOTTED1-like homeobox genes in root development [43–46], using a BLAST search with StBEL5 and POTH1 as queries, we identified several orthologues for both types in the genomes of the five root crops. (Table 2). As expected, among these TALE TFs, functional motifs like the DNA-binding domain (homeobox) and the protein/protein interactive regions (MEINOX and the SKY/BELL domains) were highly conserved. Sequence identity matches ranged from 41% in cassava and radish to 60% in sugar beet for StBEL5-like proteins and from 31% in sugar beet to 73% in carrot for POTH1-like proteins. Based on coverage and identity, the top orthologues were selected for both types for phylogenetic analyses (Figs. 3 and 4) and for scoring CU motifs in the 3´ UTR of their transcript sequences (Table 3). In mammals, such CU motifs in the RNA sequence function to interact with PTB-like proteins to regulate RNA metabolism [1, 2, 9]. In potato and pumpkin, PTB proteins bind specifically to the conserved CU motifs in the 3´ UTRs of select mobile RNAs to mediate their transport from source tissues to target sites [5, 16, 31]. The 3´ UTRs of four mobile RNAs of potato, *POTH1*, *StBEL5, − 11* and *− 29*, contain 7, 17, 7 and 11 CU motifs in their transcript sequences, respectively (Table 3; Additional file 5: Table S3). Runs of three or more CU nucleotides clustered in four sets within approximately 150 nucleotides were sufficient for binding of StPTB1 and StPTB6 to target RNAs in potato [16]. Similarly, a CU motif search in the orthologues of *StBEL*5 and *POTH1* for the five storage root crops revealed the presence of several CU motifs in the 3´ UTRs of their RNA sequences (Table 3; Additional file 5: Table S3). There are 20 such motifs in the 3´ UTR of the *POTH1-like* transcript of cassava and 15 motifs in the 3´ UTR of the *BEL5-like* transcript of sweet potato. However, 3´ UTRs of *POTH1-like* RNAs from sweet potato and radish contained only 4 and 3 CU motifs, respectively. As a negative control, two non-mobile *StBEL* mRNAs, *StBEL14* and *StBEL22*, contained only two ‘CU’ motifs each in their 3´ UTRs (Table 3; Additional file 5: Table S3).

**Table 2:** POTH1- and StBEL5-like orthologues in five storage root crops

Crops	*POTH1* (Protein ID- PGSC0003DMP400023862) (Gene ID- PGSC0003DMG400013493)				*StBEL5* (Protein ID- PGSC0003DMP400010515) (Gene ID- PGSC0003DMG400005930)			
	Orthologues		Coverage (%)	Identity (%)	Orthologues		Coverage (%)	Identity (%)
	Protein ID	Gene ID			Protein ID	Gene ID		
**Cassava** (*Manihot esculants*)	**Manes.12G025600.1** Manes.05G184900.1 Manes.13G027300.1 Manes.06G106700.1	**Manes.12G025600.1** Manes.05G184900.1 Manes.13G027300.1 Manes.06G106700.1	99 70 67 66	54 55 42 64	**Manes.09G045600.1** Manes.08G033900.1 Manes.18G021400.1	**Manes.09G045600.1** Manes.08G033900.1 Manes.18G021400.1	94 94 72	47 45 41
**Sugar beet** (*Beta vulgaris*)	**XP_010695813.1** XP_010692662.1 XP_010686774.1 XP_010686777.1 XP_010665744.1 XP_010676578.1	**104,908,392** 104,905,744 104,900,940 104,900,940 104,882,999 104,892,363	99 64 67 67 60 60	52 56 51 48 32 31	**XP_010685441.1** XP_010676226.1 XP_010672761.1	**104,899,855** 104,892,097 104,889,253	98 69 41	43 46 60
**Carrot** (*Daucus carota*)	**XP_017220366.1** XP_017248167.1 XP_017216162.1 XP_017224028.1	**108,197,298** 108,219,310 108,193,842 108,200,407	97 97 97 67	57 56 47 73	**XP_017257976.1** XP_017247149.1 XP_017227177.1 XP_017242900.1	**108,227,378** 108,218,632 108,203,009 108,215,077	96 70 41 54	45 50 58 40
**Raddish** (*Raphanus sativus*)	**XP_018442762.1** XP_018451620.1 XP_018471943.1	**108,814,646** 108,822,917 108,843,280	98 95 66	52 54 68	**XP_018483627.1** XP_018476369.1 XP_018440077.1	**108,854,534** 108,847,589 108,812,336	96 71 69	41 48 46
**Sweet potato** (*Ipomoea trifida*)	**itf15g13570.t2** itf06g23780.t1 titf09g04780.t1 itf01g32840.t1	**itf15g13570.t2|cDNA** itf06g23780.t1|cDNA itf09g04780.t1|cDNA itf01g32840.t1|cDNA	72 72 66 66	50 51 53 51	**itf04g32320.t1** itf15g01940.t1 itf09g12960.t1 itf09g01040.t1 itf05g17170.t1 itf06g21830.t2	**itf04g32320.t1|cDNA** itf15g01940.t1|cDNA itf09g12960.t1|cDNA itf09g01040.t1|cDNA itf05g17170.t1|cDNA itf06g21830.t2|cDNA	99 96 96 69 41 38	53 45 45 44 54 58

List of POTH1- and BEL5-like orthologues in storage root crops. Among the POTH1- and BEL5-like orthologues in each storage root crop, proteins identified with the best coverage and identity (highlighted in bold) were selected for constructing the dendrograms (Fig. 3, Fig. 4) and Gene IDs (bold) for scoring CU motifs in the 3´ UTRs of their respective transcripts (shown in Table 3 and Additional file 2: Table S1). Number of variants identified in each crop species as well as corresponding coverage and identity (%) for BLAST search is also provided for each orthologue

**Table 3:** Cytosine/uracil (CU) motifs in select target RNAs

Gene	Gene ID	Species	3´ UTR lengths (nt)	No. CU motifs
StBEL5*	**PGSC0003DMG400005930**	Potato	503	17
BEL5-like	**itf04g32320.t1**	*Ipomoea trifida*	428	15
BEL5-like	**Manes.09G045600.1**	Cassava	333	11
BEL5-like	**108,227,378**	Carrot	378	12
BEL5-like	**108,854,534**	Radish	316	10
BEL5-like	**104,899,855**	Sugar beet	450	14
POTH1*	**PGSC0003DMG400013493**	Potato	211	7
POTH1-like	**itf15g13570.t2**	*Ipomoea trifida*	140	4
POTH1-like	**Manes.12G025600.1**	Cassava	397	20
POTH1-like	**108,197,298**	Carrot	253	13
POTH1-like	**108,814,646**	Radish	97	3
POTH1-like	**104,908,392**	Sugar beet	248	14
StBEL11*	**PGSC0003DMG400019635**	Potato	288	7
StBEL29*	**PGSC0003DMG400021323**	Potato	329	11
StBEL14**	**PGSC0003DMG400012329**	Potato	76	2
StBEL22**	**PGSC0003DMG400022011**	Potato	74	2

The presence of cytosine/uracil (CU) motifs in the 3´ UTR of BEL5-like and POTH1-like mRNAs from a range of storage root crops. A motif was scored with at least three nucleotides containing both a cytosine and a uracil base. Among POTH1- and BEL5-like orthologues in each storage root crop, protein with the best coverage and identity (highlighted in bold) were considered for identification of CU motifs in the 3´ UTRs of respective transcript sequences (Additional file 5: Table S3). Gene IDs are given for respective orthologues. * confirmed to be a mobile mRNA, ** confirmed as non-mobile mRNAs

### Transcript detection of BEL5-, POTH1- and PTB1/6-like genes in storage root crop organs

**Fig. 5:**
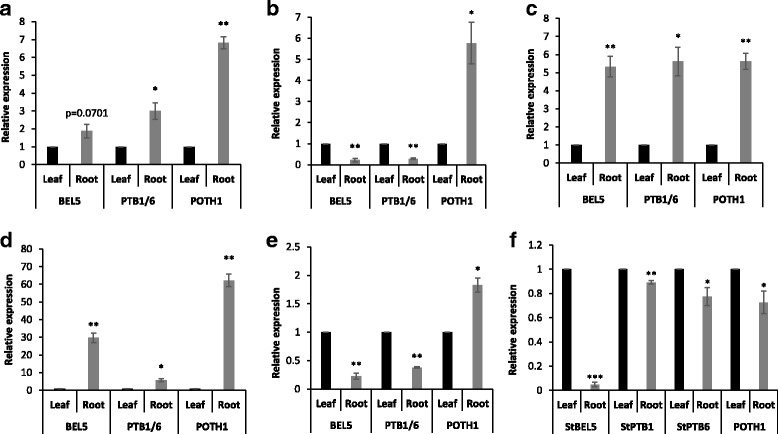
Expression analysis of StBEL5, StPTB1/6 and POTH1 orthologues in leaf and storage root samples of the root crops: sweet potato (**a**), cassava (**b**), carrot (**c**), radish (**d**) and sugar beet (**e**). Transcript levels of *StBEL5*, *StPTB1*, *StPTB6* and *POTH1* in potato leaf and root tissues are also shown from 3-month old plants (*S. tuberosum* ssp. *andigena*) grown under long-day conditions (**f**). RNA was extracted from leaves and roots and RT-qPCR with gene-specific primers was used to calculate the relative amounts of RNA for each target gene. Three biological samples were measured with three technical replicates and normalized against *GAPDH* mRNA. The fold change in RNA levels was calculated as the 2^−ΔΔCt^ value [41] relative to the mean values obtained in the leaf samples (set at a value of 1.0). Standard errors of the means are shown with one, two and three asterisks indicating significant differences (*p* < 0.05, *p* < 0.01, *p* < 0.001, respectively) using a Student’s *t*-test. Because of their close sequence match (Table 1), quantification of transcripts for PTB1 and PTB6 types in the five storage roots crops was combined as PTB1/6

To validate the activity of the conserved genes in this study, RT-qPCR with gene-specific primers was utilized to measure levels of transcripts for *POTH1*, *StBEL5*, and *StPTB1/6* orthologues in leaf and storage root samples of sweet potato, cassava, carrot, radish and sugar beet (Fig. 5a-e). Because of their close sequence match, quantification of transcripts for PTB1 and PTB6 types in the five roots crops was combined. RNAs for the orthologues were detected in both organs tested. Using RNA levels in leaves as a standard, more abundant accumulation of all three types occurred in the storage roots of sweet potato, carrot, and radish than in leaves. PTB1/6-like gene activity in storage root tissues was higher in sweet potato, carrot and radish, whereas it was lower in cassava and sugar beet, compared to leaf tissues (Fig. 5). Activity of *POTH1-like* genes was greater in storage roots than leaves in cassava and sugar beet, whereas *BEL5-like* gene activity decreased in storage roots relative to leaves in cassava, sugar beet, and potato. *StBEL5* accumulation and movement are enhanced by a short-day photoperiod [30, 31] and the potato plants used in this study were grown under long-day conditions. Except for the low level of *StBEL*5 RNA in roots, all other target genes measured here were relatively abundant in the potato organs (Fig. 5f). Overall, transcripts of the *BEL5*-, *PTB1/6*- and *POTH1*-like orthologues in the five storage root crops exhibited a significant difference in accumulation patterns in leaves compared to storage root tissues (Fig. 5).

## Discussion

### Conserved elements of the StBEL/StPTB complex in a sample of storage root crops

Comprehensive searches in the genomes of several storage root crops revealed conservation of key components in RNA/protein complexes that function in potato as regulators of both root and tuber development. Common orthologues identified include the RBPs, PTB1 and PTB6, and two mobile RNAs of potato, *StBEL5* and *POTH1*. Sequence identity matches among the proteins in the PTB1/6 family (with four RRMs) present in the several genomes searched here ranged from 72% for sugar beet to 88% for sweet potato. The four functional RRM regions and the RNPs were highly conserved in this group (Fig. 1). Several of the RNA orthologues contained an abundance of CU motifs, specific to PTB binding, in their 3´ UTRs. The existence of PTB1/6 proteins and the conserved target RNAs with their abundant CU motifs in 3´ UTRs suggest the possibility that a PTB-chaperone/*BEL*-RNA complex may be functional in the development of storage roots in a range of important crop species. It is even conceivable that PTB/RNA signal complexes are phloem mobile in these root crops.

BEL1- and KNOTTED1-type proteins are transcription factors that interact in a tandem complex to regulate gene expression. The main focus of the function of StBEL5 has been on its role as a phloem-mobile RNA signal that induces tuber formation [19, 20]. In potato, StBEL5 and its KNOX protein partner, POTH1, regulate tuberization by targeting genes that control growth [27]. Many of these genes are involved in controlling the activity and synthesis of hormones like gibberellic acid, auxin, and cytokinins. There is also solid evidence linking StBEL5 activity to root growth as well [30]. RNA movement assays with whole plants and heterografts have demonstrated that *StBEL5* transcripts move through the phloem to stolon tips, the site of tuber induction, and into roots to induce their growth [19, 30]. *StBEL5* mRNA originates in the leaf, and its movement to both stolons and roots is induced by a short-day photoperiod and mediated by two RNA-binding proteins, StPTB1 and − 6 [16]. Movement of *StBEL5* RNA to roots was correlated with increased growth, changes in morphology, and accumulation of *GA2-oxidase1*, *YUCCA1a*, and *ISOPENTENYL TRANSFERASE* transcripts [30]. Consistent with these results, a BEL1-like gene was associated with rhizome formation in lotus root and corm formation of Sagittaria [47, 48]. Three root stage-specific BEL1-like proteins were also identified in the storage roots of sweet potato [49, 50]. In addition, rhizome-specific genes of sorghum contained the *BEL5/POTH1*-specific *cis*-element, TTGAC [51], in their upstream sequence [52], suggesting that KNOX/BEL interaction with their target genes could be conserved in many plant species.

### KNOTTED1 types function in root development

In addition to the effect of POTH1 on tuber formation in potato, there are several examples documenting the role of KNOTTED1-like TFs in root and rhizome development. Four class-I KNOX genes (designated *Ibkn1–4*) were identified that were active in the storage roots of sweet potato [44, 45]. *Ibkn2* and *Ibkn4* were highly expressed in the developing storage roots of cultivars with a higher capacity for storage root formation [45]. *Ibkn1* is homologous to *SHOOT MERISTEMLESS*, whereas *Ibkn2* and *Ibkn3* are homologous to *BREVIPEDICELLUS*. Seven class-I KNOX genes have been identified in the genome of cassava [46]. Several of these KNOX genes were differentially expressed in storage root tissues suggesting they play an important role in their development. There are other examples in maize and Arabidopsis that demonstrate that KNOX genes function in root development [43, 53]. As mentioned previously, StBEL5 and its KNOX partner, POTH1, have also been linked to the regulation of root development [30, 34]. KNOX and BEL1 expression has been consistently correlated with hormone activity, specifically auxin, cytokinin, and gibberellic acid [18, 28, 30]. Movement of *StBEL5* RNA to roots was associated with enhanced growth [30, 34]. These results suggest that *StBEL5* and its transcriptional partners, like POTH1, may be involved in a developmental network that regulates hormone activity in roots through long-distance transport of *StBEL*5 mRNA [30]. In other plant species, KNOX activity is tightly linked to hormone metabolism. Using chromatin immunoprecipitation coupled with RNA-Seq profiling of KNOTTED1 (KN1) targets in maize, preferential binding of KN1 to sequence near genes belonging to the gibberellic acid, cytokinin, brassinosteroid, and auxin pathways has been demonstrated [54]. In Arabidopsis, *KNOXI* genes up-regulated cytokinin biosynthesis [55]. This is consistent with activity of the StBEL5/POTH1 complex that targets several genes involved in cytokinin metabolism including, *ISOPENTENYL TRANSFERASE*, *LONELY GUY*, and *AGL8* [27, 30]. During sweet potato storage root development, *KNOXI* gene expression and cytokinin levels were positively correlated [44]. Consistent with this observation, hormone analysis of developing storage roots of sweet potato showed that auxins, ABA, and cytokinins were involved [56].

It potato, it has been clearly demonstrated that PTB proteins, such as StPTB1 and StPTB6, and their target RNAs (e.g. *StBEL5* and *POTH1*) function as pivotal molecular signals that regulate tuber formation [16, 19, 20, 28, 29]. Moreover, *StBEL5*, *POTH1, StPTB1* and *StPTB6* genes of potato exhibit a differential pattern of expression in leaf and root tissues (Fig. 5f). Consistent with this, *BEL5*-, *PTB1/6*- and *POTH1*-like genes from five storage root crops - cassava, sweet potato, carrot, radish and sugar beet also exhibited differential patterns of expression in leaves and storage root tissues (Fig. 5). Based on our results, it is intriguing to speculate that these *BEL1-* and *POTH1-like* RNAs could function as mobile signals controlling storage organ development in root crops, with roles similar to those of the orthologues in potato.

## Conclusions

Conservation of the PTB1/6 proteins and members of the *StBEL* family was observed in genomic searches for several storage root crops. Using the RNA/PTB protein complexes of potato that function in long-distance signaling as a model, it is conceivable that similar complexes may function during storage root development. Clearly, there are limits, however, to the use of this bioinformatics approach. For example, when considering non-potato species, numerous questions arise regarding these orthologues. Are their RNAs phloem mobile? Can these PTB types mediate transcript stability and transport? Comparable to potato, can transgenic expression of the genes encoding the PTB1/6 proteins and the *BEL5-like* mRNAs affect storage root yields? Future experimental analyses will be critical to confirm the role of these components in storage root development and to assess their potential for enhancing root crop production.

## Additional files

Additional file 1: Figure S1. Phylogenetic relationship of *Ipomoea* species. (PDF 77 kb)

Additional file 2: Table S1. trnS intergenic spacer sequence analysis. (PDF 16 kb)

Additional file 3: Table S2. Gene-specific primers used for RT-qPCR. (PDF 106 kb)

Additional file 4: Figure S2. Catalog and alignments of PTB1/6 types in five root crops species. (PDF 828 kb)

Additional file 5: Table S3. Cytosine/uracil motifs in the 3´ UTRs of *BEL5-* and *POTH1-like* mRNAs. (PDF 148 kb)

## Abbreviations

AS: Alternative spicing
Bv: *Beta vulgaris*
CU: Cytosine/Uracil
Dc: *Daucus carota*
Ib: *Ipomoea batatas*
It: *Ipomoea trifida*
KN1: Knotted1-like TF
Me: *Manihot esculenta*
PTBs: Polypyrimidine binding proteins
RBPs: RNA-binding proteins
RNP: Ribonucleoprotein complex
RRMs: RNA recognition motifs
Rs: *Raphanus sativus*
TALE: Three amino acid loop extension superfamily of TFs
TFs: Transcription factors
UTRs: Untranslated regions
