# Supplementary material for: Conservation of polypyrimidine tract binding proteins and their putative target RNAs in several storage root crops
Source: BMC Genomics. 2018 Feb 7;19:124. doi: 10.1186/s12864-018-4502-7 (PMC5803842; doi:10.1186/s12864-018-4502-7)

Additional file: Figure S1. Phylogenetic relationship of *Ipomoea* species using trnS^{GCU}-trnG^{UCC} (trnS) intergenic spacer sequence derived from Table S1. Sequence was compared to other databases using NCBI BLAST. Analysis was performed using T-COFFEE (hRp://www.ch.embnet.org/soaware/TCoffee.html) and graphical representation of the phylogenetic tree was performed with TreeDyn (v198.3) [41]. Accessions for gene sequences are written after each species name in the phylogenetic tree. In the phylogenetic tree, the branch length is proportional to the number of substitutions per site and the tree is rerooted using midpoint rooting in TreeDyn.

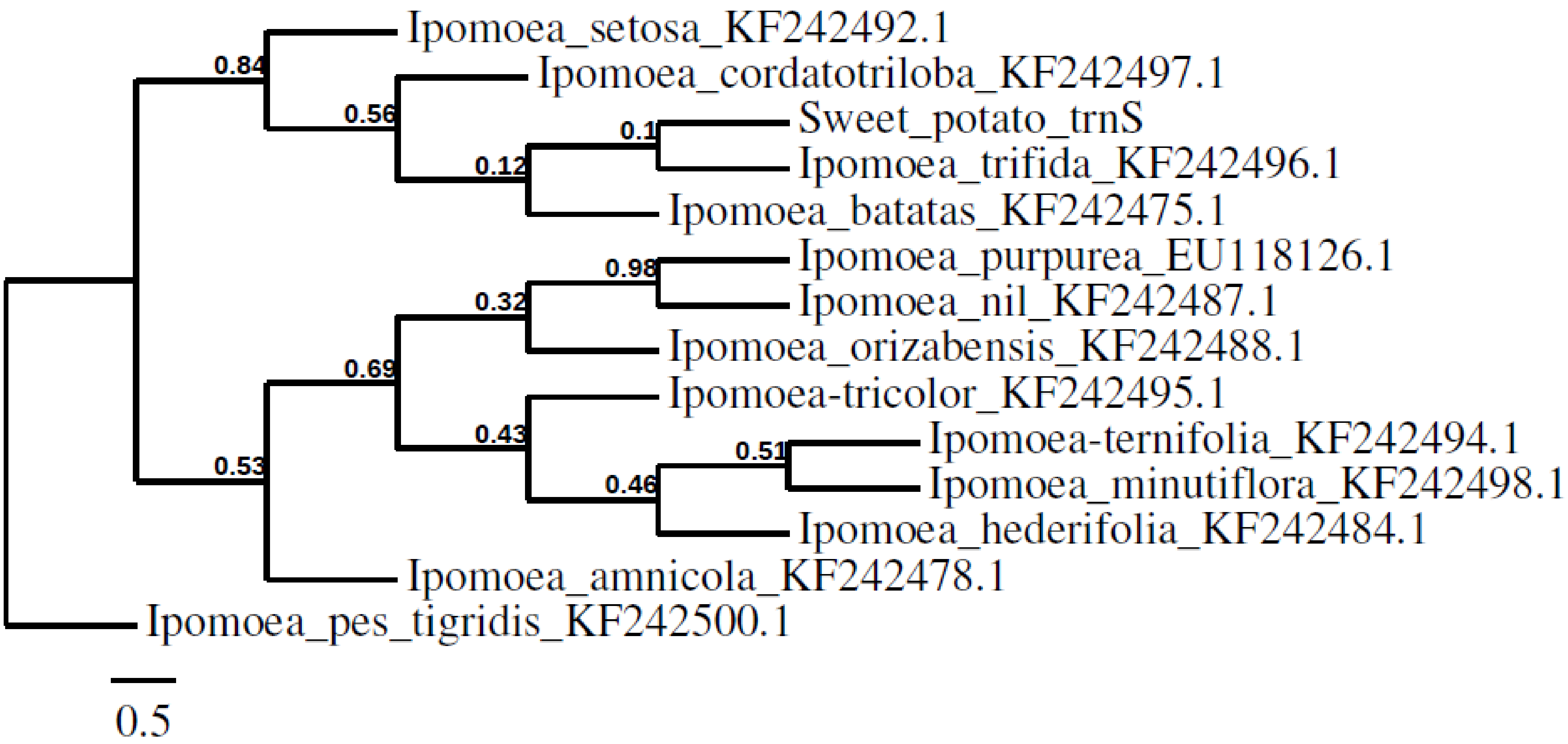

Supplement: Supplementary file 1 — Phylogenetic relationship of Ipomoea species. (PDF 77 kb) [file 12864_2018_4502_MOESM1_ESM.pdf]
