# Supplementary material for: Conservation of polypyrimidine tract binding proteins and their putative target RNAs in several storage root crops
Source: BMC Genomics. 2018 Feb 7;19:124. doi: 10.1186/s12864-018-4502-7 (PMC5803842; doi:10.1186/s12864-018-4502-7)
Supplement: Supplementary file 2 — trnS intergenic spacer sequence analysis. (PDF 16 kb) [file 12864_2018_4502_MOESM2_ESM.pdf]

Additional file: Table S1. Storage root crop species authentication through trnS^{GCU}-trnG^{UCC} (referred to as trnS-G) intergenic spacer sequence analysis.

Name	Amplicon size (bp)	Coverage (%)	Identity (%)	Species match after BLAST	Accession after BLAST search
trnS-G_sweet potato	551	100	99	*Ipomoea trifida*	KF242496.1
trnS-G_carrot	530	100	99	*Daucus carota*	KX832307.1
trnS-G_sugar beet	759	100	99	*Beta vulgaris*	KR230391.1
trnS-G_radish	655	100	92	*Raphanus sativus*	HM047361.1
trnS-G_cassava	622	100	99	*Manihot esculenta*	EU117376.1

Storage root crop species authentication by trnS^{GCU}-trnG^{UCC} (trnS-G) intergenic non-coding spacer sequence analysis

Since five storage root crops used in this study were harvested from local agriculture farm, their species authentication was required. Here, we have taken the advantage of polymorphism in chloroplast DNA regions, e.g. trnS^{GCU}-trnG^{UCC} intergenic non-coding spacer sequence (referred to as trnS-G) sequences [57-59]. Leaf genomic DNAs were isolated by DNasey Plant Mini kit (Qiagen) from leaf samples of sweet potato, cassava, carrot, radish and sugar beet. The trnS-G intergenic spacer sequences were amplified by PCR off genomic DNAs. Amplified trnS sequences from sweetpotato, carrot and sugar beet were sub-cloned into a pGEM-T Easy vector (Promega Corporation) and sequence verified. For cassava and radish, amplified PCR products of trnS-G sequences were subsequently purified and sequence verified. All the sequences obtained were subjected for global blast search using a NCBI BLAST tool to identify closely related species of storage root crops. Primers used for this analysis are listed Supplementary Table S2. The trnS-G intergenic spacer sequences were amplified by PCR from leaf genomic DNAs of sweet potato, cassava, carrot, radish and sugar beet. It was observed that amplicon size length varied in each storage root crop and was in the range of 551-759bp (Additional file: Table S1). A global blast search revealed that trnS sequence for sweetpotato matched best to *Ipomoea trifida* (accession- KF242496.1), carrot to that of *Daucus carota* (accession- KX832307.1), sugar beet to that of *Beta vulgaris* (accession- KR230391.1),

radish to that of *Raphanus sativus* (accession- HM047361.1) and cassava to that of *Manihot esculenta* (accession- EU117376.1), respectively.

References

57. Chase MW, Fay MF. Barcoding of plants and fungi. Sci. 2009; 325:682-683.

58. Holstein N, Renner SS. A dated phylogeny and collection records reveal repeated biome shifts in the African genus Coccinia (Cucurbitaceae). BMC Evolutionary Biology 2011;11:28.

59. Muellner AN, Schaefer H, Lahaye R. Evaluation of candidate DNA barcoding loci for economically important timber species of the mahogany family (Meliaceae). Mol. Ecol. Resour. 2011;11:450-460.
