# Supplementary material for: Conservation of polypyrimidine tract binding proteins and their putative target RNAs in several storage root crops
Source: BMC Genomics. 2018 Feb 7;19:124. doi: 10.1186/s12864-018-4502-7 (PMC5803842; doi:10.1186/s12864-018-4502-7)
Supplement: Supplementary file 3 — Gene-specific primers used for RT-qPCR. (PDF 106 kb) [file 12864_2018_4502_MOESM3_ESM.pdf]

Additional file: Table S2. Primers used for RT-PCR based transcript detections and real-time analysis.

Gene	Primer sequences (5'→3') Forward primer	Reverse primer	Ta (°C)	Amplicon (bp)
ItBEL5-like	CGGCGGATTCGGCGGATTC	CCGAGGGTGAGGGAAACGC	60	102
ItPTB1/6-like	GAAGGGAGGTATGCTGTTTGG	GGTGCCGTGCTCCTCCAAG	53	223
ItPOTH1-like	GAAGAAGAGGAAGAAAGGAAAG	CTTATTCTTCTCCTCTTCCG	47	109
BvBEL5-like	GGCTTATACTTAGGGATTCAA	CATCTCAACAATCCCTACTTC	44	80
BvPTB1/6-like	GTATGATGTCTGAGTGTTGGGA	CAACGGAACAGCAGACTATTAC	51	260
BvPOTH1-like	GACTAAGGATAAGCGAAAGAAATG	TCCGATGAGCCTAAAGAGTGT	47	148
DcBEL5-like	GCAAATGGGAAGAGGAGTGG	GCTGAGTGTGATGATGTTGG	49	78
DcPTB1/6-like	GTTGATGCTGGAGGAGTTTATG	GCTTATCTTCATCAATCTTATCAG	44	190
DcPOTH1-like	CGGCAGTCACATAAGTTCAC	GGTTTCCAGTGCCTCTTCCTC	49	227
RsBEL5-like	CAAGCGAAGAGACAATGATGC	GGCTCCATAATCATCTGTTTC	53	373
RsPTB1/6-like	GATATTGTTGTTCTCCTACTAAG	GCTTGCTTCTTTCCATTCATTTC	44	142
RsPOTH1-like	GTCGTCTTCTTGCTTCGGAG	CGCCTCCATTCAGTTCCTCG	51	222
MeBEL5-like	GGCATCAACACATCCACAC	GTTCTTTCTTTACACTACCAT	48	237
MePTB1/6-like	TGCTTTATCCTATTACTGTTGA	CAGAAGGAAGATGAGGATTAG	49	275
MePOTH1-like	CTGAAGCGGACAAGAAAGCAC	CAGTGAAGAAAGGTCCAGAAAG	49	153
StBEL5	CTGCAACAGCTAGGAATGATG	ATGATTTTGTCTGAATCCTTTGGG	49	137
StPTB1	GCGCAATAAACCAGATCATGC	GTTGACTTCCAGACGCTTTTC	50	112
StPTB6	TCATGCTCTTGTTCAGATGGG	TGGTCCGGTGTTAATATTTGGG	50	130
POTH1	GCTGGAAGAAATAAGGCAACA	CATCTGCCTCACCACAACTT	49	253
trnS	GCCGCTTTAGTCCACTCAGC	GAACGAATCACACTTTTACCAC	47	689
