# Supplementary material for: Conservation of polypyrimidine tract binding proteins and their putative target RNAs in several storage root crops
Source: BMC Genomics. 2018 Feb 7;19:124. doi: 10.1186/s12864-018-4502-7 (PMC5803842; doi:10.1186/s12864-018-4502-7)
Supplement: Supplementary file 4 — Catalog and alignments of PTB1/6 types in five root crops species. (PDF 828 kb) [file 12864_2018_4502_MOESM4_ESM.pdf]

Additional file: Figure S2. Catalog of PTB1/6 types in five root crops species. Red highlight is PTB1 and -6 sequence from potato, designated StPTB1 and StPTB6. Alignments for each species are shown on pp. 11-19. Yellow highlight indicates conserved RNPs.

Cassava

>StPTB1 (441aa)
MSDPSKVVHVRNVGHEISENDLLQLFQPFGVITKLVMLRAKNQALLQMQDVPSAVKALQFYSNVQPSIRGRNVYVQFSSHQELTTMDQNAQGRGDEP
NRILLVTIHHMLYPITVDVLHQVFSPHGFVEKIVTFQKSAGFQALIQYQVQQSSVSARNSLQGRNIYDGCCQLDIQFSNLDELQVNYNNERSRDYTNPNL
PSEQKGKSSQQGYGDMYSFQGSGAHPGGFPQMGNAEAIAAAFAGGLPPGISGINDRCTILVSNLNSDRINEDKLFNLCSLYGNIVSIKILRNKPDHALVQ
LGDGFQAELAVHFLKGAMLFEKRLEVNFSKYPNITTGPDTHDYSNSNLNRFNRNAAKNYRYCCSPTKMIHLSSLPQDVTEAEIIAHLEEHGPIINSKLFE
MNGKQQALVLFDKEEQATEALVCKNATSLGSSTIRISFSQLQSI*

>StPTB6 (444aa)
MTEPSKVIHVRNVGQEISENDLLQLFQPFGVITKLVMLRAKNQALLQMQDIAAAVNAMQFYSNVQPSIRGRSVYVQFSSHQELTTVDQNAQGRGDEP
NRILLVSIHHVLYPITVEVLHQVFSPHGIVEKIVTFQKSAGFQALIQYELTQTAISARNSLQGRNIYDGCCQLDIQFSNLDELQVSYNNERSRDFTNPNLPS
EPKGKSPQQGYGDAGAMYPWQGSGPRGVGFPQMGNAAAIATAFPSGLPPGISGTNDRCTIIVSNVNPDRIDEDKLFNLFSIYGNIVRIKHLRNKPDHAL
VQMGDGFQAELAVHFLKGAMLFGQRLEVNYSKYPNINTGPETRDYSNSNLNRFNRNAAKNYRYCCSPTKMIHVSSLHQDVTEEEIVAHLEEHGPIVNT
KLFEMNGKKQALILFNNEEQATEALVCQHATSLGGSIIRISFSQVQSI

>Manes.18G093400.1 (444aa)
MAELSKVIHVRNVGHEISENDLLQLFQPFGIITKLVMLRAKNQALLQMQDVASAMNALQFYSNVQPTIRGRNVYVQFSSHQELTTMDQNSQGRGDEP
NRILLVTIHHMLYPITVEVLHQVFSPHGFVEKIVTFQKSAGFQALIQYQLRQSAVAARTSLQGRNIYDGCCQLDIQFSNLDELQVNYNNDRSRDFTNPHL
PSEQKGRSSQQAGYGDVGVAYPQVGIAQVAMANAAAIAAAFGGGLPPGISGTNDRCTVLVSNLNPDRIDEDKLFNLFSLYGNIVRIKLLHNKPDHALV
QMGDGFQAELAVHFLKGAMLFGKRLEVNFSKHPNITQGADTHEYSNSNLNRFNRNAAKNYKYCCSPTKMIHLSTLPQDITEEEIVSHLEDHGAIVNTK
LFEMNGKKQALVLFETEEQATEALVCKHASSLAGSIIRISFSQLQSIRETS*

>Manes.18G093400.2 (437aa)
MAELSKVIHVRNVGHEISENDLLQLFQPFGIITKLVMLRAKNQALLQMQDVASAMNALQFYSNVQPTIRGRNVYVQFSSHQELTTMDQNSQGRGDEP
NRILLVTIHHMLYPITVEVLHQVFSPHGFVEKIVTFQKSAGFQALIQYQLRQSAVAARTSLQGRNIYDGCCQLDIQFSNLDELQVNYNNDRSRDFTNPHL
PSEQKGRSSQQAGYGDVGVAYPQMANAAAIAAAFGGGLPPGISGTNDRCTVLVSNLNPDRIDEDKLFNLFSLYGNIVRIKLLHNKPDHALVQMGDGFQ
AELAVHFLKGAMLFGKRLEVNFSKHPNITQGADTHEYSNSNLNRFNRNAAKNYKYCCSPTKMIHLSTLPQDITEEEIVSHLEDHGAIVNTKLFEMNGK
KQALVLFETEEQATEALVCKHASSLAGSIIRISFSQLQSIRETS*

>Manes.18G093400.3 (436aa)
MAELSKVIHVRNVGHEISENDLLQLFQPFGIITKLVMLRAKNQALLQMQDVASAMNALQFYSNVQPTIRGRNVYVQFSSHQELTTMDQNSQGRGDEP
NRILLVTIHHMLYPITVEVLHQVFSPHGFVEKIVTFQKSAGFQALIQYQLRQSAVAARTSLQGRNIYDGCCQLDIQFSNLDELQVNYNNDRSRDFTNPHL
PSEQKGRSSQAGYGDVGVAYPQMANAAAIAAAFGGGLPPGISGTNDRCTVLVSNLNPDRIDEDKLFNLFSLYGNIVRIKLLHNKPDHALVQMGDGFQA

ELAVHFLKGAMLFGKRLEVNFSKHPNITQGADTHEYSNSNLNRFNRNAAKNYKYCCSPTKMIHLSTLPQDITEEEIVSHLEDHGAIVNTKLFEMNGKK
QALVLFETEEQATEALVCKHASSLAGSIIRISFSQLQSIRETS*

>Manes.02G181600.1 (437aa)
MTEPSKVIHVRNVGHEISENDLLQLFQPFGVITKLVMLRSKNQALLQMQDIPSAINALQFYSNVQPTIRGRNVYVQFSSHQELTTMDQNSQGLGDEPNR
ILLVTIHHMLYPITVEVLHQVFSPHGFVEKIVTFQKSAGFQALIQYQLCQSAVAARTALQGRNIYDGCCQLDIQFSNLDELQVNYNNDRSRDFTNPHLPS
EQKGRTSQPAGYGDVGVAYPQMANATAIAAAFGGGLPPGISGTNDRCTVLVSNLNLDRVDEDKLFNLFSLYGNIVRIKFLRNKPDHALVQMGDGFQA
ELAVHFLKGAMLFGKRLEVNFSKHPNITQGADTHEYSNSNLNRFNRNAAKNYRYCCSPTKMIHLSTLPQDISEEEIVSHLEEHGTIVNTKLFEMNGKKQ
ALVLFETEEQATEAVVCKHASSLAGSIIRISFSQLQSIRETS*

Note: .1, .2, and .3 are same except .1 has 7/6 additional amino acids- ‘GIAQVAM’

Distribution of the top 5 Blast Hits on 5 subject sequences

Mouse over to see the title, click to show alignments

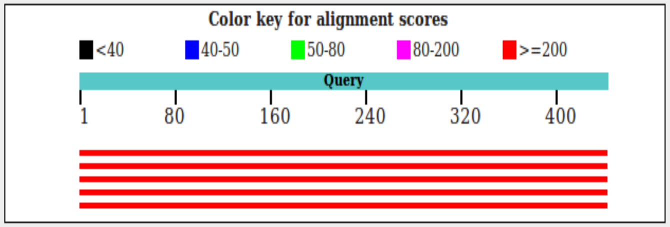

Descriptions

Sequences producing significant alignments:

Select: All None Selected:0

Alignments Download Graphics Distance tree of results Multiple alignment

Description	Max score	Total score	Query cover	E value	Ident	Accession
StPTB6	788	788	99%	0.0	84%	Query_27775
Manes.18G093400.3 (436aa)	765	765	99%	0.0	84%	Query_27778
Manes.18G093400.2 (437aa)	763	763	99%	0.0	84%	Query_27777
Manes.02G181600.1 (437 aa)	760	760	99%	0.0	84%	Query_27779
Manes.18G093400.1 (444aa)	756	756	99%	0.0	83%	Query_27776

Alignments

Sugar beet
>StPTB1
MSDPSKVVHVRNVGHEISENDLLQLFQPFGVITKLVMLRAKNQALLQMQDVPSAVKALQFYSNVQPSIRGRNVYVQFSSHQELTTMDQNAQGRGDEP
NRILLVTIHHMLYPITVDVLHQVFSPHGFVEKIVTFQKSAGFQALIQYQVQQSSVSARNSLQGRNIYDGCCQLDIQFSNLDELQVNYNNERSRDYTNPNL
PSEQKGKSSQQGYGDMYSFQGSGAHPGGFPQMGNAEAIAAAFAGGLPPGISGINDRCTILVSNLNSDRINEDKLFNLCSLYGNIVSIKILRNKPDHALVQ
LGDGFQAELAVHFLKGAMLFEKRLEVNFSKYPNITTGPDTHDYSNSNLNRFNRNAAKNYRYCCSPTKMIHLSSLPQDVTEAEIIAHLEEHGPIINSKLFE
MNGKQQALVLFDKEEQATEALVCKNATSLGSSTIRISFSQLQSI*

>StPTB6
MTEPSKVIHVRNVGQEISENDLLQLFQPFGVITKLVMLRAKNQALLQMQDIAAAVNAMQFYSNVQPSIRGRSVYVQFSSHQELTTVDQNAQGRGDEP
NRILLVSIHHVLYPITVEVLHQVFSPHGIVEKIVTFQKSAGFQALIQYELTQTAISARNSLQGRNIYDGCCQLDIQFSNLDELQVSYNNERSRDFTNPNLPS
EPKGKSPQQGYGDAGAMYPWQGSGPRGVGFPQMGNAAAIATAFPSGLPPGISGTNDRCTIIVSNVNPDRIDEDKLFNLFSIYGNIVRIKHLRNKPDHAL
VQMGDGFQAELAVHFLKGAMLFGQRLEVNYSKYPNINTGPETRDYSNSNLNRFNRNAAKNYRYCCSPTKMIHVSSLHQDVTEEEIVAHLEEHGPIVNT
KLFEMNGKKQALILFNNEEQATEALVCQHATSLGGSIIRISFSQVQSI

>XP_010681101.1 PREDICTED: polypyrimidine tract-binding protein homolog 3 [Beta vulgaris subsp. vulgaris]
MSEASKVIHVRNVGHEISENDLLQLFQPFGVITKLVMLRAKNQALVQMQDVPSAVNILQYYTNTQPTIRGRNVYIQFSSHQELTTMDQNSHGRGDEPN
RILLVTIHQLVYPITVDVLHQVFSPHGLVEKIVTFQKSAGFQALIQYQSRHSAVTARNLLQGRNIYDGCCQLDIQYSNLDELQVNYNNERTRDFTNSSLP
SEPKGRSSQPGYADGGGMYPLPAGGSPVAFPQMANHSAIAAAFGGVFPPGITGMNDRCTVLVSNLDPDRIDEDKLFNLFSLYGNIVRIKLLRNKPDHAL
VQMGDGFQAELAVHFLKGITLFGKRLDVNYSRYPQITTGADTHEYLNSNLNRFNRNAAKNYRYCCSPTKMIHLSTIPQDITEEEIVSHLEEHGTIVSTKV
FETNGKKQALVLFEDEEQATEALVCKNATTLDGSVIRISFSQSQAI

>XP_010680298.1 PREDICTED: polypyrimidine tract-binding protein homolog 3 [Beta vulgaris subsp. vulgaris]
MSETSKVIHVRNVGHEITENDLLQLVQPFGSVTKLVMLRTKNQALLQMQDVATAINLVDYYTNVQPNVRGRNVYMQFSSHQELTTDQTQGRKSDTD
GQPNRILLVSIHHVIYPMTVDVLNQVFSPYGFVEKVVTFQKSAGYQALVQYQTRQSAASAMSALHGRNIYDGCCQLDVQYSNLTELQVNSNNDRSRD
FTNPNLPSEQRGRSSQSGYGDAGGLFPFQPTGSGAVAYGQMGNAAAMAAFSGGLPPGVSGTNDRCTVIVSNLNPDRIDEDKLFNLFSLYGNIVRIKLLR
GKPDHALVEMSDGFQAELAVHFLKGAILFGKKIEVNFSKYSNITPSPDTRDYQNSNLNRFNHNAAKHYKYCCSPTKMIHLSSVPEDVSEEEIVELIEEHG
QVVNTKVFEANGKKQALVQFETEEQATEALVSKHATSVCGSIVRISFSQSQST

>XP_010693257.1 PREDICTED: polypyrimidine tract-binding protein homolog 1 isoform X2 [Beta vulgaris subsp. vulgaris]
MSTSGQHQFRYTQTPSKVLHLRNLPWECSEEELVELCKPFGKIVNTKCNVGANRNQAFVEYADLNQAISMVSYYASASEPAQVRGKTVYIQYSNRHEI
VNNKSPGDIPGNVLLVTMEGVQPGDVTIEVIHLVFSAFGFVHKIATFEKAAGFQALIQFSDVDTASAAKNALDGRSIPRYLLPEHVSSCNLRISYSAHTD
LNIKFQSHRSRDYTNPYLPVNPTAFDGLSQPVVGADGVKKEQESNVLLAAVENMQYAVTVDVLHTVFSAFGTVQKIAIFEKNGQTQALIQYPDVSTAS
VAKDSLEGHCIYDGGYCS

>XP_010671511.1 PREDICTED: polypyrimidine tract-binding protein homolog 2 isoform X1 [Beta vulgaris subsp. vulgaris]
MASVSSQPQFRYTQPPSKVLHLRNLPWECTEEELIELGKPFGKVVNTKCNVGANKNQAFIEFADLNQAIAMISYYASSSEPAQIRGKTVYLQYSNRQEI
VNNKTTADTAGNVLLVTIEGNEARQVSIEVLHLVFSAFGFVHKITTFEKTAGFQALIQFSDTETASSAKNALDGRSIPRYLLPDHVGPCTLRITYSAHTDL
SVKFQSHRSRDYTNPYLPVAPSAIDATGQISVGVDGKKLEPESNVLLASIENMQYAVTLDVLQTVFSAFGVVQKIAMFDKNGGLQALIQYPDVQTAVV

AKEALEGHCIYDGGFCKLHLSYSRHTDLSIKVNNDRSRDYTIPIHAVIQPSSILGQQPTGPMPGPGAPPPYNGGQYPGPPSSGGWGAPPQGPHHMQMPM
QNYQYMPPGAAPPGPPGTMPPGSAPPGSMQPGSMHMQNPNGLPQPPAMHPYSQ

>KMT16075.1 hypothetical protein BVRB_3g052430 isoform B [Beta vulgaris subsp. vulgaris]
MASVSSQPQFRYTQPPSKVLHLRNLPWECTEEELIELGKPFGKVVNTKCNVGANKNQAFIEFADLNQAIAMISYYASSSEPAQIRGKTVYLQYSNRQEI
VNNKTTADTAGNVLLVTIEGNEARQVSIEVLHLVFSAFGFVHKITTFEKTAGFQALIQFSDTETASSAKNALDGRSIPRYLLPDHVGPCTLRITYSAHTDL
SVKFQSHRSRDYTNPYLPVAPSAIDATGQISVGVDGKKLEPESNVLLASIENMQYAVTLDVLQTVFSAFGVVQKIAMFDKNGGLQALIHVHVSQEQLL
VMLCDVQTAVVAKEALEGHCIYDGGFCKLHLSYSRHTDLSIKVNNDRSRDYTIPIHAVIQPSSILGQQPTGPMPGPGAPPPYNGGQYPGPPSSGGWGAP
PQGPHHMQMPMQNYQYMPPGAAPPGPPGTMPPGSAPPGSMQPGSMHMQNPNGLPQPPAMHPYSQ

>XP_010693256.1 PREDICTED: polypyrimidine tract-binding protein homolog 1 isoform X1 [Beta vulgaris subsp. vulgaris]
MSTSGQHQFRYTQTPSKVLHLRNLPWECSEEELVELCKPFGKIVNTKCNVGANRNQAFVEYADLNQAISMVSYYASASEPAQVRGKTVYIQYSNRHEI
VNNKSPGDIPGNVLLVTMEGVQPGDVTIEVIHLVFSAFGFVHKIATFEKAAGFQALIQFSDVDTASAAKNALDGRSIPRYLLPEHVSSCNLRISYSAHTD
LNIKFQSHRSRDYTNPYLPVNPTAFDGLSQPVVGADGVKKEQESNVLLAAVENMQYAVTVDVLHTVFSAFGTVQKIAIFEKNGQTQALIQYPDVSTAS
VAKDSLEGHCIYDGGYCKLHLTYSRHTDLNVKAHSDKSRDYTIPEIVQQTSGYPGAPAVPWMNPQTGAGYPPNGYSTNANMPPQTHAAPAPSWDPS
MQPGRRTFVSVPSTFPGQTYTASPVPAYASAPISPAPGASMPMTPPAASRASQAPYYP

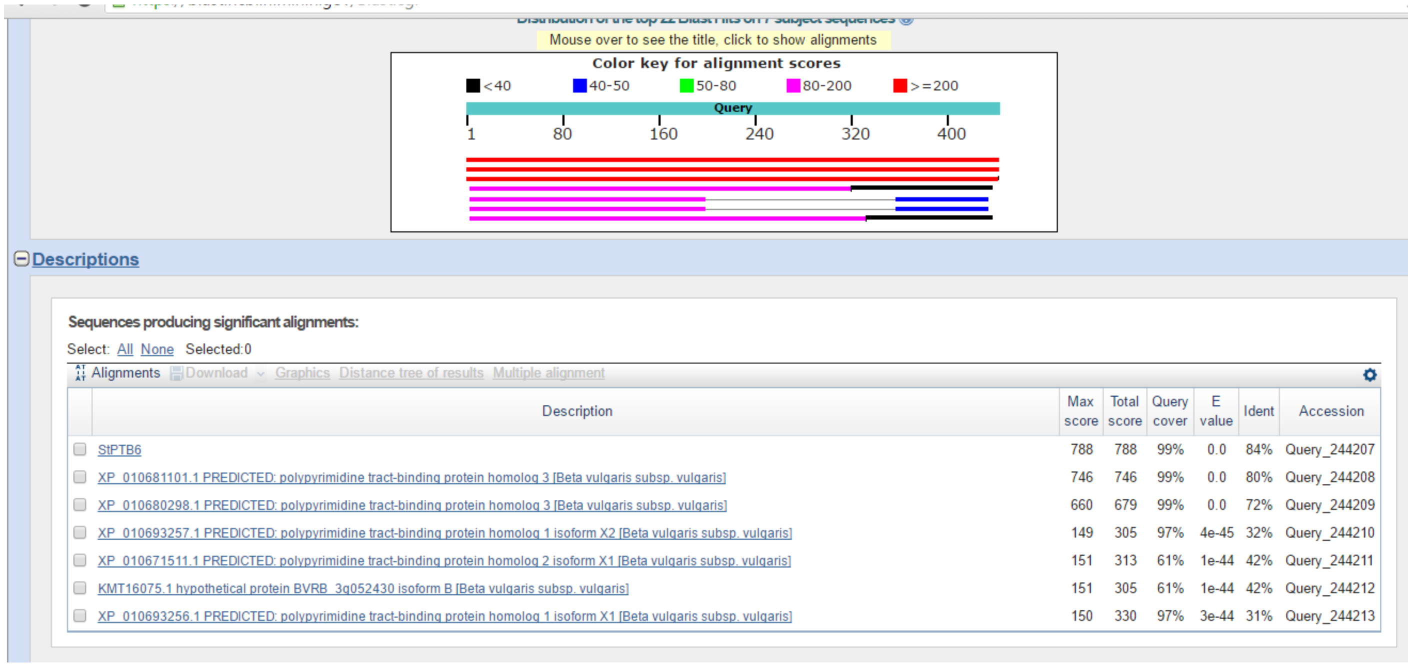

Description	Max score	Total score	Query cover	E value	Ident	Accession
StPTB6	788	788	99%	0.0	84%	Query_244207
XP_010681101.1 PREDICTED: polypyrimidine tract-binding protein homolog 3 [Beta vulgaris subsp. vulgaris]	746	746	99%	0.0	80%	Query_244208
XP_010680298.1 PREDICTED: polypyrimidine tract-binding protein homolog 3 [Beta vulgaris subsp. vulgaris]	660	679	99%	0.0	72%	Query_244209
XP_010693257.1 PREDICTED: polypyrimidine tract-binding protein homolog 1 isoform X2 [Beta vulgaris subsp. vulgaris]	149	305	97%	4e-45	32%	Query_244210
XP_010671511.1 PREDICTED: polypyrimidine tract-binding protein homolog 2 isoform X1 [Beta vulgaris subsp. vulgaris]	151	313	61%	1e-44	42%	Query_244211
KMT16075.1 hypothetical protein BVRB_3g052430 isoform B [Beta vulgaris subsp. vulgaris]	151	305	61%	1e-44	42%	Query_244212
XP_010693256.1 PREDICTED: polypyrimidine tract-binding protein homolog 1 isoform X1 [Beta vulgaris subsp. vulgaris]	150	330	97%	3e-44	31%	Query_244213

Carrot
>StPTB1
MSDPSKVVHVRNVGHEISENDLLQLFQPFGVITKLVMLRAKNQALLQMQDVPSAVKALQFYSNVQPSIRGRNVYVQFSSHQELTTMDQNAQGRGDEP
NRILLVTIHHMLYPITVDVLHQVFSPHGFVEKIVTFQKSAGFQALIQYQVQQSSVSARNSLQGRNIYDGCCQLDIQFSNLDELQVNYNNERSRDYTNPNL
PSEQKGKSSQQGYGDMYSFQGSGAHPGGFPQMGNAEAIAAAFAGGLPPGISGINDRCTILVSNLNSDRINEDKLFNLCSLYGNIVSIKILRNKPDHALVQ
LGDGFQAELAVHFLKGAMLFEKRLEVNFSKYPNITTGPDTHDYSNSNLNRFNRNAAKNYRYCCSPTKMIHLSSLPQDVTEAEIIAHLEEHGPIINSKLFE
MNGKQQALVLFDKEEQATEALVCKNATSLGSSTIRISFSQLQSI*

>StPTB6
MTEPSKVIHVRNVGQEISENDLLQLFQPFGVITKLVMLRAKNQALLQMQDIAAAVNAMQFYSNVQPSIRGRSVYVQFSSHQELTTVDQNAQGRGDEP
NRILLVSIHHVLYPITVEVLHQVFSPHGIVEKIVTFQKSAGFQALIQYELTQTAISARNSLQGRNIYDGCCQLDIQFSNLDELQVSYNNERSRDFTNPNLPS
EPKGKSPQQGYGDAGAMYPWQGSGPRGVGFPQMGNAAAIATAFPSGLPPGISGTNDRCTIIVSNVNPDRIDEDKLFNLFSIYGNIVRIKHLRNKPDHAL
VQMGDGFQAELAVHFLKGAMLFGQRLEVNYSKYPNINTGPETRDYSNSNLNRFNRNAAKNYRYCCSPTKMIHVSSLHQDVTEEEIVAHLEEHGPIVNT
KLFEMNGKKQALILFNNEEQATEALVCQHATSLGGSIIRISFSQVQSI

>XP_017247842.1 PREDICTED: polypyrimidine tract-binding protein homolog 3 [Daucus carota subsp. sativus]
MAEPSKVIHVRNVGHEISENDLLQLFQPFGVITKLVMLRAKNQALLQMQDVTTAVNALQFYTNVQPSIRGRNVYIQFSSHQELTTVEQNAQGRGDEPN
RILLVTIHHMLYPITVEVLHQVFSPHGFVEKIVTFQKSAGFQALIQFQLKQSAVTARNSLQGRNIYDGCCQLDIQFSNLDELQVNYNNERSRDFTNPSLPA
EQRGRSSQPGYVDAGGVYGFQPSGVRPVGFPQMDNAAAIAAAFGGGLPPGITGTNDRCTILVSNLNTDKIDEDKLFNLFSLYGNIVRIKLLRNKPDHAL
VQMGDGFQAELAVHFLKGATLFEKRLEVNFSKHPNITTGTETHEYSNSNLNRFNRNAAKNYRYCCSPTKMIHLSTLPQDVTEEEIVTHLEEHGTIANTK
VFDMNGKKQALVMFETEEQATEALVCKHATSLGGQMIRISFSQLQNI

>XP_017241710.1 PREDICTED: polypyrimidine tract-binding protein homolog 1 [Daucus carota subsp. sativus]
MSNPNQPQFRYTQTPSKVLHLRNLPWECIEEELVELCKPFGKIVNTKCNVGANRNQAFVEFVELNQAINMVSYYASSSEPASIRGKHVYIQYSNRHEIV
NNKGPGDVPGNVLLVTIEGVEAGDVSIDVIHLVFSAFGFVHKIATFEKAAGFQALIQFTDAETALSAREALDGRSIPRYLLPEHVGSCNLRISYSAHTDLN
IKFQSHRSRDYTNPYLPVNSTAIEGFVQPVVGPDGKKKEPESNVLLASIENRIYDVTVDVLNTVFSAFGTVQKIAIFEKNATTQALIQYPDINIAAAAKDA
LEGHCIYDGGYCKLHISYSRHTDLNVKAFSDKSRDYTVPESGFAAALPAGATVWQNPHAAPVFTASEFVGVNYAQPQGPHGHVNYMQPLQGPPGQGP
PGQGPPGQGPPGQGPPGQGPPGQAPSWDPAMQLSGPSFVSGSSTLPGQTYGPTSGQVYNPMGSPAGSSPYGPPSGQVYNPMSSPAGSSPLGQKTQVNPS
SSASGGQPGNPSNMQHGSSSPRNHHPPYYR

>XP_017242965.1 PREDICTED: polypyrimidine tract-binding protein homolog 2 [Daucus carota subsp. sativus]
MASVSSQPQFRYTQPPSKVLHLRNLPWECTEDELIELGKPFGKVVNTKCNVGANRNQAFIEFAELNQAIAMISYYASSSEPAQVRGKTVYLQYSNRQEI
VNNKTTADVAGNVLLVTIEGNDARLVSIEVLHVVFSAFGFVHKITTFEKTAGFQALVQFTDSETASSAKDALDGRSIPSYLIPELAPCSLRITYSAHTDLS
VKFQSHRSRDYTNPNLPVNPSAIDATGQISKGLDGKKLEPESNVLLASIENMPYEVTLEVLHMVFSAFGTVLKIAMFDKNGGVQALIQYPDIQTAVVAK
EALEGHCIYDGGYCKLHISYSRHTDLSIKVNNDRSRDYTIPMSSVLSTQPSILGQLPPAMVSSGVPQYNGSHYASAHQGHPVHPPSSGWSAGPPAVPQP
MPGQMHNPQYMPPASMPSEYGHQMMHSPNGFQNAGTYPRYPPQ

>XP_017246841.1 PREDICTED: polypyrimidine tract-binding protein homolog 2-like [Daucus carota subsp. sativus]
MSSVSSQPQFRYTQPPSKVLHLRNLPWECTEEELIELGKPFGKVVNTKCNVGANRNQAFIEFAELNQAIAMISYFASSSEAAQVRGKTVYLQYSNRQEI
VNNKTTADVAGNVLLVTIEGNDARLVSIEVLHVVFSAFGFVHKITTFEKTAGFQALVQFTDSETASSAKDALDGRSIPSYLIPELSPCSLKITYSAHTDLS

VKFQSHRSRDYTNPSLPVNPSAIDVTGQISMGLDGKRLEPESNVLLASIENMPYELTLDVLHMVFSTFGTVLKIAMFDKNGGIQALVQYPDVQTAVVA
KQALEGHCVYDGGYCKLHISFSRHTDLSIKVNNNRSRDYTIPNVPLLSTQPSMLAQQSPSLLGPGGPQYNATQFAPVHEGQAMPQPPSGWNSGAPAGP
QPMQGQMHPHHYMPANMPSEYGHSVMHNPNSFHHAGPYPHYPPQ

>KZM98104.1 hypothetical protein DCAR_014534 [Daucus carota subsp. sativus]
MGGQQRIKKYILGPDISLVSTVLCLDFVIVESVMSSVSSQPQFRYTQPPSKVLHLRNLPWECTEEELIELGKPFGKVVNTKCNVGANRNQAFIEFAELNQ
AIAMISYFASSSEAAQVRGKTVYLQYSNRQEIVNNKTTADVAGNVLLVTIEGNDARLVSIEVLHVVFSAFGFVHKITTFEKTAGFQALVQFTDSETASSA
KDALDGRSIPSYLIPELSPCSLKITYSAHTDLSVKFQSHRSRDYTNPSLPVNPSAIDVTGQISMGLDGKRLEPESNVLLASIENMPYELTLDVLHMVFSTFG
TVLKIAMFDKNGGIQALVQYPDVQTAVVAKQALEGHCVYDGGYCKLHISFSRHTDLSIKVNNNRSRDYTIPNVPLLSTQPSMLAQQSPSLLGPGGPQY
NATQFAPVHEGQAMPQPPSGWNSGAPAGPQPMQGQMHPHHYMPANMPSEYGHSVMHNPNSFHHAGPYPHYPPQ

>KZN02333.1 hypothetical protein DCAR_011087 [Daucus carota subsp. Sativus]
MSNPNQPQFRYTQTPSKVLHLRNLPWECIEEELVELCKPFGKIVNTKCNVGANRNQAFVEFVELNQAINMVSYYASSSEPASIRGKHVYIQYSNRHEIV
NNKGPGDVPGNVLLVTIEGVEAGDVSIDVIHLVFSAFGFVHKIATFEKAAGFQALIQFTDAETALSAREALDGRSPDGKKKEPESNVLLASIENRIYDVT
VDVLNTVFSAFGTVQKIAIFEKNATTQALIQYPDINIAAAAKDALEGHCIYDGGYCKLHISYSRHTDLNVKAFSDKSRDYTVPESGFAAALPAGATVWQ
NPHAAPVFTASEFVGVNYAQPQGPHGHVNYMQPLQGPPGQGPPGQGPPGQGPPGQGPPGQGPPGQAPSWDPAMQLSGPSFVSGSSTLPGQTYGPTSG
QVYNPMGSPAGSSPYGPPSGQVYNPMSSPAGSSPLGQKTQVNPSSSASGGQPGNPSNMQHGSSSPRNHHPPYYR

Distribution of the top 24 Blast Hits on 7 subject sequences

Mouse over to see the title, click to show alignments

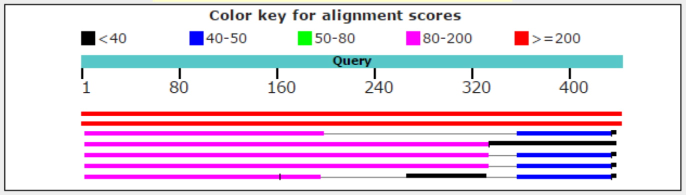

Descriptions

Sequences producing significant alignments:

Select: All None Selected:0

Alignments Download Graphics Distance tree of results Multiple alignment

Description	Max score	Total score	Query cover	E value	Ident	Accession
StPTB6	788	788	99%	0.0	84%	Query_719
XP_017247842.1 PREDICTED: polypyrimidine tract-binding protein homolog 3 [Daucus carota subsp. sativus]	786	786	99%	0.0	85%	Query_720
XP_017241710.1 PREDICTED: polypyrimidine tract-binding protein homolog 1 [Daucus carota subsp. sativus]	156	331	62%	8e-46	43%	Query_721
XP_017242965.1 PREDICTED: polypyrimidine tract-binding protein homolog 2 [Daucus carota subsp. sativus]	147	313	98%	5e-43	30%	Query_722
XP_017246841.1 PREDICTED: polypyrimidine tract-binding protein homolog 2-like [Daucus carota subsp. sativus]	142	313	92%	3e-41	30%	Query_723
KZM98104.1 hypothetical protein DCAR_014534 [Daucus carota subsp. sativus]	142	314	92%	5e-41	30%	Query_724
KZN02333.1 hypothetical protein DCAR_011087 [Daucus carota subsp. Sativus]	127	307	76%	9e-36	43%	Query_725

Radish

>StPTB1
MSDPSKVVHVRNVGHEISENDLLQLFQPFGVITKLVMLRAKNQALLQMQDVPSAVKALQFYSNVQPSIRGRNVYVQFSSHQELTTMDQNAQGRGDEP
NRILLVTIHHMLYPITVDVLHQVFSPHGFVEKIVTFQKSAGFQALIQYQVQQSSVSARNSLQGRNIYDGCCQLDIQFSNLDELQVNYNNERSRDYTNPNL
PSEQKGKSSQQGYGDMYSFQGSGAHPGGFPQMGNAEAIAAAFAGGLPPGISGINDRCTILVSNLNSDRINEDKLFNLCSLYGNIVSIKILRNKPDHALVQ
LGDGFQAELAVHFLKGAMLFEKRLEVNFSKYPNITTGPDTHDYSNSNLNRFNRNAAKNYRYCCSPTKMIHLSSLPQDVTEAEIIAHLEEHGPIINSKLFE
MNGKQQALVLFDKEEQATEALVCKNATSLGSSTIRISFSQLQSI*

>StPTB6
MTEPSKVIHVRNVGQEISENDLLQLFQPFGVITKLVMLRAKNQALLQMQDIAAAVNAMQFYSNVQPSIRGRSVYVQFSSHQELTTVDQNAQGRGDEP
NRILLVSIHHVLYPITVEVLHQVFSPHGIVEKIVTFQKSAGFQALIQYELTQTAISARNSLQGRNIYDGCCQLDIQFSNLDELQVSYNNERSRDFTNPNLPS
EPKGKSPQQGYGDAGAMYPWQGSGPRGVGFPQMGNAAAIATAFPSGLPPGISGTNDRCTIIVSNVNPDRIDEDKLFNLFSIYGNIVRIKHLRNKPDHAL
VQMGDGFQAELAVHFLKGAMLFGQRLEVNYSKYPNINTGPETRDYSNSNLNRFNRNAAKNYRYCCSPTKMIHVSSLHQDVTEEEIVAHLEEHGPIVNT
KLFEMNGKKQALILFNNEEQATEALVCQHATSLGGSIIRISFSQVQSI

>XP_018451916.1 PREDICTED: polypyrimidine tract-binding protein homolog 3 [Raphanus sativus]
MAESSKVIHVRNVGHEISENDLLQLFQPFGVITKLVMLRAKNQALLQMQDVSSAITALQFFTTVQPTIRGRNVYIQFSSHQELTTAEQNIHGREDEPNRI
LLVTVHHMLYPITVDVLHQVFSPYGFVEKIVTFQKSAGFQALIQYQAQQCAASARTSLQGRNIYDGCCQLDIQFSNLEELQVNYNNDRSRDYTNPNLPS
EQKGRLPHPGYGDAGVAYPQMANTSAIAAAFGGGLPPGITGTNDRCTILVSNLNTDSVDEDKLFNLFSLYGNIVRIKLLRNKPDHALVQMGDGFQAEL
AVHFLKGAMLFGKRLEVNYSKHPNITPGTDSHDYVNSNLNRFNRNAAKNYRYCCSPTKMIHLSTLPQDVTEEEVVNHVQEHGAILNTKVFEMNGKKQ
ALVQFENEEEAAEALVCKHATSLGGSIIRISFSQLQTI

>XP_018441449.1 PREDICTED: polypyrimidine tract-binding protein homolog 1-like isoform X1 [Raphanus sativus]
MSSSSQFRYTQTPSKVVHLRNLPWECVEEELIDLCKRFGKIVNTKTNVGANRNQAFVEFAEVNQAISMVSYYASSSEPAQIRGKTVYIQYSNRHEIVNN
QSPGEVPGNVLLVTFEGVESHHVSIDVIHLVFSAYGFVHKIATFEKAAGFQALVQFTDVDTALAARTALDGRSIPKYLLPEHVGSCNLRMSYSAHTDLN
IKFQSHRSRDYTDPYLPVNQTAMDGSMQPALGADGKRVETQSNVLLALIENMQYAVTVDVLHTVFSAYGTVQKIAIFEKNGSTQALIQYSDIPTATIAK
EALEGHCIYDGGYCKLRLTYSRHTDLNVKAFSDKSRDYTLPDLSQLVGQKVPGVAAASGPTDGWHNGQVQTQYAGSSYMYSPADPTGASPSSGHPPY
YG

>XP_018441450.1 PREDICTED: polypyrimidine tract-binding protein homolog 1-like isoform X2 [Raphanus sativus]
MSSSSQFRYTQTPSKVVHLRNLPWECVEEELIDLCKRFGKIVNTKTNVGANRNQAFVEFAEVNQAISMVSYYASSSEPAQIRGKTVYIQYSNRHEIVNN
QSPGEVPGNVLLVTFEGVESHHVSIDVIHLVFSAYGFVHKIATFEKAAGFQALVQFTDVDTALAARTALDGRSIPKYLLPEHVGSCNLRMSYSAHTDLN
IKFQSHRSRDYTDPYLPVNQTAMDGSMQPALGADGKRVETQSNVLLALIENMQYAVTVDVLHTVFSAYGTVQKIAIFEKNGSTQALIQYSDIPTATIAK
EALEGHCIYDGGYCI

>XP_018472559.1 PREDICTED: polypyrimidine tract-binding protein homolog 2 [Raphanus sativus]
MSSVSSQQQFRYTQTPSKVLHLRNLPWECTEEELIELGKPFGTVVNTKCNVGANKNQAFIEFEDLNQAIQMISFYASSSEPAQVRGKTVYLQYSNRQEI
VNNKTAADVVGNVLLVTVEGEDARMVSIDVLHLVFSAFGFVHKITTFEKTAGYQALVQFTDAETATSARTSLDGRNIPSYLLPEEVSPCSLKITYSAHT
DLTVKFQSHRSRDYTNPYLPVAPSAIDSTGQVVVGVDGRKMEPESNVLLASIENMQYAVTLDVLHTVFVAFGAVQKIAMFDKNGGVQALIQYPDVQT

AVVAKGALEGHCIYEGGFCKLHITYSRHTDLSIKVNNDRSRDYTMPDPAVAMAPQPGHNPYPSNSPQYQGADASHYQQQQPQGGWGQQSGGQGHNP
YMGGPPSMHQGGPGGYMPPHHYGSGPSGPMH

>XP_018488837.1 PREDICTED: polypyrimidine tract-binding protein homolog 1 isoform X2 [Raphanus sativus]
MSSSQFRYTQTPSKVVHLRNLPWECVEEELIDLCKRFGKIVNTKTNVGANRNQAFVEFAELNQAISMVSYYASSSEPAQIRGKTIYIQYSNRHEIVNNQS
PGEVPGNVLLVTFEGVESHHVSIDVIHLVFSAFGFVHKIATFEKAAGFQALVQFTDVETASAARSALDGRSIPKYLLPEHVASCNLRMSYSAHTDLNIKF
QSHRSRDYTNPYLPVNHTAMDGSMQPALGADGKKVETQSNVLLALIENMQYAVTVDVLHTVFSAYGTVQKIAIFEKNGSTQALIQYSDIATAAIAKEA
LEGHCIYDGGYCKLRLSYSRHTDLNVKVHKIGCFLELWERD

>XP_018488838.1 PREDICTED: polypyrimidine tract-binding protein homolog 1 isoform X3 [Raphanus sativus]
MSSSQFRYTQTPSKVVHLRNLPWECVEEELIDLCKRFGKIVNTKTNVGANRNQAFVEFAELNQAISMVSYYASSSEPAQIRGKTIYIQYSNRHEIVNNQS
PGEVPGNVLLVTFEGVESHHVSIDVIHLVFSAFGFVHKIATFEKAAGFQALVQFTDVETASAARSALDGRSIPKYLLPEHVASCNLRMSYSAHTDLNIKF
QSHRSRDYTNPYLPVNHTAMDGSMQPALGADGKKVETQSNVLLALIENMQYAVTVDVLHTVFSAYGTVQKIAIFEKNGSTQALIQYSDIATAAIAKEA
LEGHCIYDGGYCI

>XP_018488836.1 PREDICTED: polypyrimidine tract-binding protein homolog 1 isoform X1 [Raphanus sativus]
MSSSQFRYTQTPSKVVHLRNLPWECVEEELIDLCKRFGKIVNTKTNVGANRNQAFVEFAELNQAISMVSYYASSSEPAQIRGKTIYIQYSNRHEIVNNQS
PGEVPGNVLLVTFEGVESHHVSIDVIHLVFSAFGFVHKIATFEKAAGFQALVQFTDVETASAARSALDGRSIPKYLLPEHVASCNLRMSYSAHTDLNIKF
QSHRSRDYTNPYLPVNHTAMDGSMQPALGADGKKVETQSNVLLALIENMQYAVTVDVLHTVFSAYGTVQKIAIFEKNGSTQALIQYSDIATAAIAKEA
LEGHCIYDGGYCKLRLSYSRHTDLNVKAFSDKSRDYTLPDLSLLVGQKVPGVAEASAPTTGGWQNGQVQTQYAGYGGSSYMYPSADPTGASPSSGHP
PYYG

>XP_018463656.1 PREDICTED: polypyrimidine tract-binding protein homolog 1-like [Raphanus sativus]
MSSSQFRYTQTPSKVVHLRNLPWECVEEELIDLCKRFGKIVNTKTNVGANRNQAFVEFAELNQAISMVSYYASSSEPAQIRGKTIYIQYSNRHEIVNNQS
PGEVPGNVLLVTFEGVESHHLSIDVIHLVFSAFGFVHKIATFEKAAGFQALVQFTDVETASAARSALDGRSIPKYLLPEHVASCNLRMSYSAHTDLNIKF
QSHRSRDYTNPYLPVNHTAMDGSMQPSLGADGKKVETQSNVLLALIENMQYAVTVDVLHTVFSAYGTVQKIAIFEKNGSTQALIQYSDIATAAIAKEA
LEGHCIYDGGYCKLRLSYSRHTDLNVKAFSDKSRDYTLPDLSLLVGQKVPGVAEASAPTTGGWQNGQVQTQYAGYGGSPYMYPSADPTGASPSSGHP
PYYG

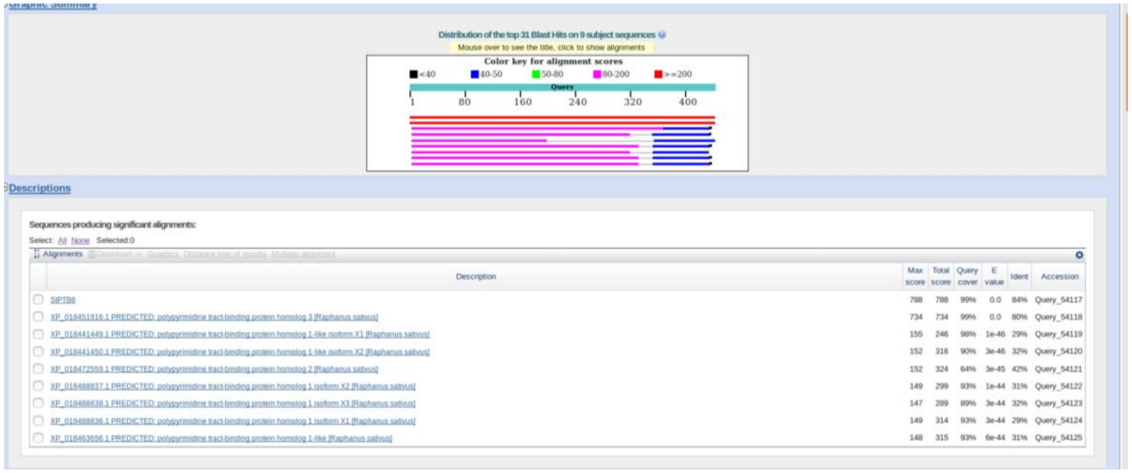

Description	Max score	Total score	Query cover	E value	Ident	Accession
SlPTB6	788	788	99%	0.0	84%	Query_54117
XP_018451916.1 PREDICTED: polypyrimidine tract-binding protein homolog 3 [Raphanus sativus]	734	734	99%	0.0	80%	Query_54118
XP_018441449.1 PREDICTED: polypyrimidine tract-binding protein homolog 1-like isoform X1 [Raphanus sativus]	155	246	98%	1e-46	29%	Query_54119
XP_018441450.1 PREDICTED: polypyrimidine tract-binding protein homolog 1-like isoform X2 [Raphanus sativus]	152	316	90%	3e-46	32%	Query_54120
XP_018472559.1 PREDICTED: polypyrimidine tract-binding protein homolog 2 [Raphanus sativus]	152	324	64%	3e-45	42%	Query_54121
XP_018488837.1 PREDICTED: polypyrimidine tract-binding protein homolog 1 isoform X2 [Raphanus sativus]	149	299	93%	1e-44	31%	Query_54122
XP_018488838.1 PREDICTED: polypyrimidine tract-binding protein homolog 1 isoform X3 [Raphanus sativus]	147	289	89%	3e-44	32%	Query_54123
XP_018488836.1 PREDICTED: polypyrimidine tract-binding protein homolog 1 isoform X1 [Raphanus sativus]	149	314	93%	3e-44	29%	Query_54124
XP_018463656.1 PREDICTED: polypyrimidine tract-binding protein homolog 1-like [Raphanus sativus]	148	315	93%	6e-44	31%	Query_54125

Sweet potato (*I. trifida*)

>StPTB1
MSDPSKVVHVRNVGHEISENDLLQLFQPFGVITKLVMLRAKNQALLQMQDVPSAVKALQFYSNVQPSIRGRNVYVQFSSHQELTTMDQNAQGRGDEP
NRILLVTIHHMLYPITVDVLHQVFSPHGFVEKIVTFQKSAGFQALIQYQVQQSSVSARNSLQGRNIYDGCCQLDIQFSNLDELQVNYNNERSRDYTNPNL
PSEQKGKSSQQGYGDMYSFQGSGAHPGGFPQMGNAEAIAAAFAGGLPPGISGINDRCTILVSNLNSDRINEDKLFNLCSLYGNIVSIKILRNKPDHALVQ
LGDGFQAELAVHFLKGAMLFEKRLEVNFSKYPNITTGPDTHDYSNSNLNRFNRNAAKNYRYCCSPTKMIHLSSLPQDVTEAEIIAHLEEHGPIINSKLFE
MNGKQQALVLFDKEEQATEALVCKNATSLGSSTIRISFSQLQSI*

>StPTB6
MTEPSKVIHVRNVGQEISENDLLQLFQPFGVITKLVMLRAKNQALLQMQDIAAAVNAMQFYSNVQPSIRGRSVYVQFSSHQELTTVDQNAQGRGDEP
NRILLVSIHHVLYPITVEVLHQVFSPHGIVEKIVTFQKSAGFQALIQYELTQTAISARNSLQGRNIYDGCCQLDIQFSNLDELQVSYNNERSRDFTNPNLPS
EPKGKSPQQGYGDAGAMYPWQGSGPRGVGFPQMGNAAAIATAFPSGLPPGISGTNDRCTIIVSNVNPDRIDEDKLFNLFSIYGNIVRIKHLRNKPDHAL
VQMGDGFQAELAVHFLKGAMLFGQRLEVNYSKYPNINTGPETRDYSNSNLNRFNRNAAKNYRYCCSPTKMIHVSSLHQDVTEEEIVAHLEEHGPIVNT
KLFEMNGKKQALILFNNEEQATEALVCQHATSLGGSIIRISFSQVQSI

>itf09g10450.t1
MTEPSKVIHVRNVGHEISENDLLQLFQPFGVITKLVMLRAKNQALLQMQDVPSAVNALQFYTNVQPSIRGRNVYIQFSSHQELTTMDQNTQSRGDEPN
RILLVTIHHMLYPITVEVLHQVFSPHGFVEKIVTFQKSAGFQALIQYQANQSAVSARNALQGRNIYDGCCQLDIQFSNLDELQVNYNNDRSRDFTNPNLP
SEQKGKSSQHGYGDAGGMYSLQAPVGFPQMGNAAAIAAAFPGGLPPGISGTNDRCTILVSNLNPDRIDEDKLFNLFSIYGNIVRIKLLRNKPDHALVQM
GDGFQAELAVHFLKGGMLFGKKLEVNFSKHPNITTGPDTHDYSNSNLNRFNRNAAKNYRYCCSPTKMIHLSTLPQDVSETEIVAHLEEHGTIVNSKLFE
MNGKKQALVLFENEEQATEALVCKNASSLGGSTIRISFSQLQNI*

>itf05g19430.t1
MSSVSSQPQFRYTQPPSKVLHLRNLPWECTEEELIELGKPFGRVVNTKCNVGANRNQAFIEFAEQNQAIAMISYYASSSEPAQVRGKTVYLQYSNRQEI
VNNKTTADVAGNVLLVTIEGNDARLVSIDILHLVFSAFGFVHKITTFEKTAGFQALVQFSDAETATSAKDALDGRSIPSYLIPDLGPCSLKITYSAHTDLS
VKFQSHRSRDYTNPHLPVAPSAIDASGQFTVGLDGKKLDPESNVLLAAIENMQYAVTVDVLHTVFSAFGPVLKIAMFDKNGGLQALVQYPDVQTAVA
AKDALEGHCIYEGGFCKLHITYSRHTDLSIKVNNDRGRDYTIPNAPMSSQPPVLLGQQQSPTGGPGAHPYNATQYASAPNVHAAPQAASSWNFVGAA
GPPPMSMQMHNPPYMGPANVHNQNRPAMPPYQLR*

>itf05g16040.t1
MSTSGQPQFRYTQTPSKVLHLRNLPWECIEEELVELCKPFGRIVNSKCNVGSNRNQAFVEFADLNQAINMATYYASSSEPAQIRGKTVYIQYSNRHEIV
NNKSPGDIPGNVLLVTIEGVEAGDVSIDVIHLVFSAFGFVHKIATFEKAAGFQALVQFTDIETAATAREALDGRSIPRYLLPQHVSNCHLRISFSAHTDLNI
KFQSNRSRDYTNPYLPVNPTAMEGFVQPVVGADGKKKEPESNVLLASIENMQYAVTVDVLHTVFSAFGTVQKIAIFEKNGGTQAFIQYPDVTTATVAK
DALEGHCIYDGGYCKLHLSYSRHTDLNVKAYSDKSRDYRVPESGLPPLQQASALPNTAPAWQNHPQYVSVHSAPPATMPMQNPWDPTMQGGRPAFIS
APTTFPGQEYASPPVPAYTTAAPIPPGSSPTSQPNAPFGASHPAHQGIPPPGQPR*

>itf12g25960.t1
MSSVSSQPQFRYTQPPSKVLHLRNLPWECSEEELIELGKPFGKVVNTKCNVGANRNQAFIEFAELNQAIAMISYYASSSEPAQVRGKTVYLQYSNRQEI
VNNKTTADVAGNVLLVTIEGNDARLVSIDVLHLVFSAFGFVHKITTFEKTAGFQALVQFSDAVTATSAKDALDGRSIPSYLIPELGPCSLRITYSAHTDLT
VKFQSHRSRDYTNPLLPVAPSAIDANSQFSVGLDGKKLEPESNVLLASIENMQYAVTLDVLHTVFAAFGPVLKIAMFDKNGGLQALIQYPDVQTAVVA

KEALEGHSIYEGGYCKLHITYSRHTDLSIKVNNDRSRDYTIPNAPMLNAQPSNMGGQQPPPSVGGPGGHPYNGNQYAPAHDGHAAPQPSASWNSGNV
GGPPPMGMQMHNSPYMPSASGPQGYNQSGLPHSGAMPPYQPR*

>itf05g19430.t2
MSSVSSQPQFRYTQPPSKVLHLRNLPWECTEEELIELGKPFGRVVNTKCNVGANRNQAFIEFAEQNQAIAMISYYASSSEPAQVRGKTVYLQYSNRQEI
VNNKTTADVAGNVLLVTIEGNDARLVSIDILHLVFSAFGFVHKITTFEKTAGFQALVQFSDAETATSAKDALDGRSIPSYLIPDLGPCSLKITYSAHTDLS
VKDYTNPHLPVAPSAIDASGQFTVGLDGKKLDPESNVLLAAIENMQYAVTVDVLHTVFSAFGPVLKIAMFDKNGGLQALVQYPDVQTAVAAKDALE
GHCIYEGGFCKLHITYSRHTDLSIKVNNDRGRDYTIPNAPMSSQPPVLLGQQQSPTGGPGAHPYNATQYASAPNVHAAPQAASSWNFVGAAGPPPMSM
QMHNPPYMGPANVHNQNRPAMPPYQLR*

Distribution of the top 21 Blast Hits on 6 subject sequences

Mouse over to see the title, click to show alignments

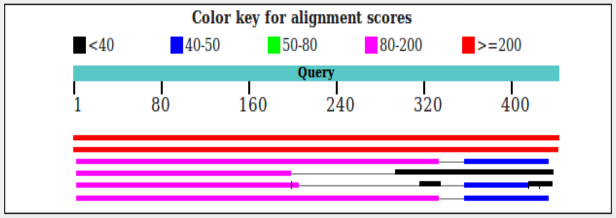

escriptions

Sequences producing significant alignments:

Select: All None Selected:0

Alignments Download Graphics Distance tree of results Multiple alignment

	Description	Max score	Total score	Query cover	E value	Ident	Accession
	itf09g10450.t1	804	804	100%	0.0	88%	Query_207629
	StPTB6	788	788	99%	0.0	84%	Query_207628
	itf05g19430.t1	147	361	91%	2e-43	31%	Query_207630
	itf12g25960.t1	146	325	76%	5e-43	41%	Query_207632
	itf05g16040.t1	146	341	68%	6e-43	41%	Query_207631
	itf05g19430.t2	134	317	91%	1e-38	30%	Query_207633

Cassava PTB1/6 types alignment (StPTB1/6 conserved RRMs in red and RNPs in yellow)

```
StPTB1               MSDPSKVVHVRNVGHEISENDLLQLFQPFGVITKLVMLRAKNQALLQMQDVPSAVKALQF   60
StPTB6               MTEPSKVIHVRNVGQEISENDLLQLFQPFGVITKLVMLRAKNQALLQMQDIAAAVNAMQF   60
Manes.18G093400.1    MAELSKVIHVRNVGHEISENDLLQLFQPFGIITKLVMLRAKNQALLQMQDVASAMNALQF   60
Manes.18G093400.2    MAELSKVIHVRNVGHEISENDLLQLFQPFGIITKLVMLRAKNQALLQMQDVASAMNALQF   60
Manes.18G093400.3    MAELSKVIHVRNVGHEISENDLLQLFQPFGIITKLVMLRAKNQALLQMQDVASAMNALQF   60
Manes.02G181600.1    MTEPSKVIHVRNVGHEISENDLLQLFQPFGVITKLVMLRSKNQALLQMQDIPSAINALQF   60
                     *:: ***:******:***************:********:**********: :*::*:**

StPTB1               YSNVQPSIRGRNVYVQFSSHQELTTMDQNAQGRGDEPNRILLVTIHHMLYPITVDVLHQV   120
StPTB6               YSNVQPSIRGRSVYVQFSSHQELTTVDQNAQGRGDEPNRILLVSIHHVLYPITVEVLHQV   120
Manes.18G093400.1    YSNVQPTIRGRNVYVQFSSHQELTTMDQNSQGRGDEPNRILLVTIHHMLYPITVEVLHQV   120
Manes.18G093400.2    YSNVQPTIRGRNVYVQFSSHQELTTMDQNSQGRGDEPNRILLVTIHHMLYPITVEVLHQV   120
Manes.18G093400.3    YSNVQPTIRGRNVYVQFSSHQELTTMDQNSQGRGDEPNRILLVTIHHMLYPITVEVLHQV   120
Manes.02G181600.1    YSNVQPTIRGRNVYVQFSSHQELTTMDQNSQGLGDEPNRILLVTIHHMLYPITVEVLHQV   120
                     ******:****.*************:***:** **********:***:******:*****

StPTB1               FSPHGFVEKIVTFQKSAGFQALIQYQVQQSSVSARNSLQGRNIYDGCCQLDIQFSNLDEL   180
StPTB6               FSPHGIVEKIVTFQKSAGFQALIQYELTQTAISARNSLQGRNIYDGCCQLDIQFSNLDEL   180
Manes.18G093400.1    FSPHGFVEKIVTFQKSAGFQALIQYQLRQSAVAARTSLQGRNIYDGCCQLDIQFSNLDEL   180
Manes.18G093400.2    FSPHGFVEKIVTFQKSAGFQALIQYQLRQSAVAARTSLQGRNIYDGCCQLDIQFSNLDEL   180
Manes.18G093400.3    FSPHGFVEKIVTFQKSAGFQALIQYQLRQSAVAARTSLQGRNIYDGCCQLDIQFSNLDEL   180
Manes.02G181600.1    FSPHGFVEKIVTFQKSAGFQALIQYQLCQSAVAARTALQGRNIYDGCCQLDIQFSNLDEL   180
                     *****:*******************:: *::::**.:***********************

StPTB1               QVNYNNERSRDYTNPNLPSEQKGKSSQQ-GYGDMYSF-QGSGAHPGG--FPQMGNAEAIA   236
StPTB6               QVSYNNERSRDFTNPNLPSEPKGKSPQQ-GYGDAGAMYPWQGSGPRGVGFPQMGNAAAIA   239
Manes.18G093400.1    QVNYNNDRSRDFTNPHLPSEQKGRSSQQAGYGDVGVA-----YPQVGIAQVAMANAAAIA   235
Manes.18G093400.2    QVNYNNDRSRDFTNPHLPSEQKGRSSQQAGYGDVGVA-----YPQM-------ANAAAIA   228
Manes.18G093400.3    QVNYNNDRSRDFTNPHLPSEQKGRSS-QAGYGDVGVA-----YPQM-------ANAAAIA   227
Manes.02G181600.1    QVNYNNDRSRDFTNPHLPSEQKGRTSQPAGYGDVGVA-----YPQM-------ANATAIA   228
                     **.***:****:***:**** **::    ****                    .** ***

StPTB1               AAFAGGLPPGISGINDRCTILVSNLNSDRINEDKLFNLCSLYGNIVSIKILRNKPDHALV   296
StPTB6               TAFPSGLPPGISGTNDRCTIIVSNVNPDRIDEDKLFNLFSIYGNIVRIKHLRNKPDHALV   299
Manes.18G093400.1    AAFGGGLPPGISGTNDRCTVLVSNLNPDRIDEDKLFNLFSLYGNIVRIKLLHNKPDHALV   295
Manes.18G093400.2    AAFGGGLPPGISGTNDRCTVLVSNLNPDRIDEDKLFNLFSLYGNIVRIKLLHNKPDHALV   288
Manes.18G093400.3    AAFGGGLPPGISGTNDRCTVLVSNLNPDRIDEDKLFNLFSLYGNIVRIKLLHNKPDHALV   287
Manes.02G181600.1    AAFGGGLPPGISGTNDRCTVLVSNLNLDRVDEDKLFNLFSLYGNIVRIKFLRNKPDHALV   288
                     :** .******** *****::***:* **::******* *:***** ** *:********

StPTB1               QLGDGFQAELAVHFLKGAMLFEKRLEVNFSKYPNITTGPDTHDYSNSNLNRFNRNAAKNY   356
StPTB6               QMGDGFQAELAVHFLKGAMLFGQRLEVNYSKYPNINTGPETRDYSNSNLNRFNRNAAKNY   359
Manes.18G093400.1    QMGDGFQAELAVHFLKGAMLFGKRLEVNFSKHPNITQGADTHEYSNSNLNRFNRNAAKNY   355
Manes.18G093400.2    QMGDGFQAELAVHFLKGAMLFGKRLEVNFSKHPNITQGADTHEYSNSNLNRFNRNAAKNY   348
Manes.18G093400.3    QMGDGFQAELAVHFLKGAMLFGKRLEVNFSKHPNITQGADTHEYSNSNLNRFNRNAAKNY   347
Manes.02G181600.1    QMGDGFQAELAVHFLKGAMLFGKRLEVNFSKHPNITQGADTHEYSNSNLNRFNRNAAKNY   348
                     *:******************* :*****:**:***. * :*::*****************
```

```
StPTB1                 RYCCSPTKMIHLSSLPQDVTEAEIIAHLEEHGPIINSKLFEMNGKQQALVLFDKEEQATE   416
StPTB6                 RYCCSPTKMIHVSSLHQDVTEEEIVAHLEEHGPIVNTKLFEMNGKKQALILFNNEEQATE   419
Manes.18G093400.1      KYCCSPTKMIHLSTLPQDITEEEIVSHLEDHGAIVNTKLFEMNGKKQALVLFETEEQATE   415
Manes.18G093400.2      KYCCSPTKMIHLSTLPQDITEEEIVSHLEDHGAIVNTKLFEMNGKKQALVLFETEEQATE   408
Manes.18G093400.3      KYCCSPTKMIHLSTLPQDITEEEIVSHLEDHGAIVNTKLFEMNGKKQALVLFETEEQATE   407
Manes.02G181600.1      RYCCSPTKMIHLSTLPQDISEEEIVSHLEEHGTIVNTKLFEMNGKKQALVLFETEEQATE   408
                       :**********:*:* **::* **::***:** *:*:********:***:**:.******

StPTB1                 ALVCKNATSLGSSTIRISFSQLQSI*----    441
StPTB6                 ALVCQHATSLGGSIIRISFSQVQSI-----    444
Manes.18G093400.1      ALVCKHASSLAGSIIRISFSQLQSIRETS*    444
Manes.18G093400.2      ALVCKHASSLAGSIIRISFSQLQSIRETS*    437
Manes.18G093400.3      ALVCKHASSLAGSIIRISFSQLQSIRETS*    436
Manes.02G181600.1      AVVCKHASSLAGSIIRISFSQLQSIRETS*    437
                       *:**::*:**..* *******:***
```

Sugar beet PTB1/6 types alignment

```
StPTB1              ------------MSDPSKVVHVRNVGHEISENDLLQLFQPFGVIT--KLVMLRAKNQALL     46
StPTB6              ------------MTEPSKVIHVRNVGQEISENDLLQLFQPFGVIT--KLVMLRAKNQALL     46
XP_010681101.1      ------------MSEASKVIHVRNVGHEISENDLLQLFQPFGVIT--KLVMLRAKNQALV     46
XP_010680298.1      ------------MSETSKVIHVRNVGHEITENDLLQLVQPFGSVT--KLVMLRTKNQALL     46
XP_010693257.1      -MSTSGQHQFRYTQTPSKVLHLRNLPWECSEEELVELCKPFGKIVNTKCNVGANRNQAFV     59
XP_010671511.1      MASVSSQPQFRYTQPPSKVLHLRNLPWECTEEELIELGKPFGKVVNTKCNVGANKNQAFI     60
KMT16075.1          MASVSSQPQFRYTQPPSKVLHLRNLPWECTEEELIELGKPFGKVVNTKCNVGANKNQAFI     60
XP_010693256.1      -MSTSGQHQFRYTQTPSKVLHLRNLPWECSEEELVELCKPFGKIVNTKCNVGANRNQAFV     59
                                    ***:*:**:  * :*::*::* :*** :.  *  :   :***::

StPTB1              QMQDVPSAVKALQFYSNV--QPSIRGRNVYVQFSSHQELTTMDQNA--QGRGDEPNRILL     102
StPTB6              QMQDIAAAVNAMQFYSNV--QPSIRGRSVYVQFSSHQELTTVDQNA--QGRGDEPNRILL     102
XP_010681101.1      QMQDVPSAVNILQYYTNT--QPTIRGRNVYIQFSSHQELTTMDQNS--HGRGDEPNRILL     102
XP_010680298.1      QMQDVATAINLVDYYTNV--QPNVRGRNVYMQFSSHQELTTDQTQGRKSDTDGQPNRILL     104
XP_010693257.1      EYADLNQAISMVSYYASASEPAQVRGKTVYIQYSNRHEIVNNKS------PGDIPGNVLL     113
XP_010671511.1      EFADLNQAIAMISYYASSSEPAQIRGKTVYLQYSNRQEIVNNKT------TADTAGNVLL     114
KMT16075.1          EFADLNQAIAMISYYASSSEPAQIRGKTVYLQYSNRQEIVNNKT------TADTAGNVLL     114
XP_010693256.1      EYADLNQAISMVSYYASASEPAQVRGKTVYIQYSNRHEIVNNKS------PGDIPGNVLL     113
                    :  *:  *:  :.:*:.      :**:.**:*:*.::*:.. .          .  ..:**

StPTB1              VTIHHM-LYPITVDVLHQVFSPHGFVEKIVTFQKSAGFQALIQYQVQQSSVSARNSLQGR     161
StPTB6              VSIHHV-LYPITVEVLHQVFSPHGIVEKIVTFQKSAGFQALIQYELTQTAISARNSLQGR     161
XP_010681101.1      VTIHQL-VYPITVDVLHQVFSPHGLVEKIVTFQKSAGFQALIQYQSRHSAVTARNLLQGR     161
XP_010680298.1      VSIHHV-IYPMTVDVLNQVFSPYGFVEKVVTFQKSAGYQALVQYQTRQSAASAMSALHGR     163
XP_010693257.1      VTMEGVQPGDVTIEVIHLVFSAFGFVHKIATFEKAAGFQALIQFSDVDTASAAKNALDGR     173
XP_010671511.1      VTIEGNEARQVSIEVLHLVFSAFGFVHKITTFEKTAGFQALIQFSDTETASSAKNALDGR     174
KMT16075.1          VTIEGNEARQVSIEVLHLVFSAFGFVHKITTFEKTAGFQALIQFSDTETASSAKNALDGR     174
XP_010693256.1      VTMEGVQPGDVTIEVIHLVFSAFGFVHKIATFEKAAGFQALIQFSDVDTASAAKNALDGR     173
                    *::.      ::::*:: *** .*:*.*:.**:*:**:***:*:.  .:: :* . *.**

StPTB1              NIY-------DGCCQLDIQFSNLDELQVNYNNERSRDYTNPNLPSEQKGKSSQQ--GYGD     212
StPTB6              NIY-------DGCCQLDIQFSNLDELQVSYNNERSRDFTNPNLPSEPKGKSPQQ--GYGD     212
XP_010681101.1      NIY-------DGCCQLDIQYSNLDELQVNYNNERTRDFTNSSLPSEPKGRSSQP--GYAD     212
```

```
XP_010680298.1      NIY-------DGCCQLDVQYSNLTELQVNSNNDRSRDFTNPNLPSEQRGRSSQS--GYGD      214
XP_010693257.1      SIPRYLLPEHVSSCNLRISYSAHTDLNIKFQSHRSRDYTNPYLPVNPTAFDGLSQPVVGA      233
XP_010671511.1      SIPRYLLPDHVGPCTLRITYSAHTDLSVKFQSHRSRDYTNPYLPVAPSAIDATGQISVGV      234
KMT16075.1          SIPRYLLPDHVGPCTLRITYSAHTDLSVKFQSHRSRDYTNPYLPVAPSAIDATGQISVGV      234
XP_010693256.1      SIPRYLLPEHVSSCNLRISYSAHTDLNIKFQSHRSRDYTNPYLPVNPTAFDGLSQPVVGA      233
                    .*         . * * : :*   :*.:. :..*:**:**  **    . .       .

StPTB1              ---MYSFQGSGAHPGGFPQMGNAEAIAAAFAGGLPPGISGINDRCTILVSNLNSDRINED      269
StPTB6              AGAMYPWQGSGPRGVGFPQMGNAAAIATAFPSGLPPGISGTNDRCTIIVSNVNPDRIDED      272
XP_010681101.1      GGGMYPLPAG-GSPVAFPQMANHSAIAAAFGGVFPPGITGMNDRCTVLVSNLDPDRIDED      271
XP_010680298.1      AGGLFPFQPTGSGAVAYGQMGNAAAM-AAFSGGLPPGVSGTNDRCTVIVSNLNPDRIDED      273
XP_010693257.1      DGV------------KKEQES----------NVLLAAVENM------------QYAVTVD      259
XP_010671511.1      DGK------------KLEPES----------NVLLASIENM------------QYAVTLD      260
KMT16075.1          DGK------------KLEPES----------NVLLASIENM------------QYAVTLD      260
XP_010693256.1      DGV------------KKEQES----------NVLLAAVENM------------QYAVTVD      259
                                        .          . :  .: .                :  *

StPTB1              KLFNLCSLYGNIVSIKILRNKPD-HALVQ-----------LGDGFQAELAVHFLKGAMLF      317
StPTB6              KLFNLFSIYGNIVRIKHLRNKPD-HALVQ-----------MGDGFQAELAVHFLKGAMLF      320
XP_010681101.1      KLFNLFSLYGNIVRIKLLRNKPD-HALVQ-----------MGDGFQAELAVHFLKGITLF      319
XP_010680298.1      KLFNLFSLYGNIVRIKLLRGKPD-HALVE-----------MSDGFQAELAVHFLKGAILF      321
XP_010693257.1      VLHTVFSAFGTVQKIAIFEKNGQTQALIQ-----------YPDVSTASVAKDSLEGHCIY      308
XP_010671511.1      VLQTVFSAFGVVQKIAMFDKNGGLQALIQ-----------YPDVQTAVVAKEALEGHCIY      309
KMT16075.1          VLQTVFSAFGVVQKIAMFDKNGGLQALIHVHVSQEQLLVMLCDVQTAVVAKEALEGHCIY      320
XP_010693256.1      VLHTVFSAFGTVQKIAIFEKNGQTQALIQ-----------YPDVSTASVAKDSLEGHCIY      308
                     * .: * :* :  *  :  :   :**:.             *   * :* . *:*  ::

StPTB1              EKR---LEVNFSKYPNITTGPDTHDYSNSNLNRFNRNAAKNYRYCCSPTKMIHLSSLPQD      374
StPTB6              GQR---LEVNYSKYPNINTGPETRDYSNSNLNRFNRNAAKNYRYCCSPTKMIHVSSLHQD      377
XP_010681101.1      GKR---LDVNYSRYPQITTGADTHEYLNSNLNRFNRNAAKNYRYCCSPTKMIHLSTIPQD      376
XP_010680298.1      GKK---IEVNFSKYSNITPSPDTRDYQNSNLNRFNHNAAKHYKYCCSPTKMIHLSSVPED      378
XP_010693257.1      DGGYCS------------------------------------------------------      314
XP_010671511.1      DGGFCKLHLSYSRHTDLSIK-------------VNNDRSRD--YTIPIHAVIQPSSILGQ      354
KMT16075.1          DGGFCKLHLSYSRHTDLSIK-------------VNNDRSRD--YTIPIHAVIQPSSILGQ      365
XP_010693256.1      DGGYCKLHLTYSRHTDLNVK-------------AHSDKSRD--YTIPEIVQQ--TSGYPG      351

StPTB1              VTEAEIIAHLEEHGP--IINSKLFEMNGKQQALV----LFDKEEQATEALVCKNATSLGS      428
StPTB6              VTEEEIVAHLEEHGP--IVNTKLFEMNGKKQALI----LFNNEEQATEALVCQHATSLGG      431
XP_010681101.1      ITEEEIVSHLEEHGT--IVSTKVFETNGKKQALV----LFEDEEQATEALVCKNATTLDG      430
XP_010680298.1      VSEEEIVELIEEHGQ--VVNTKVFEANGKKQALV----QFETEEQATEALVSKHATSVCG      432
XP_010693257.1      ------------------------------------------------------------      314
XP_010671511.1      QPTGPM----PGPGAPPPYNGGQYP--GPPS-----SGGWGAPPQGPHH-----------      392
KMT16075.1          QPTGPM----PGPGAPPPYNGGQYP--GPPS-----SGGWGAPPQGPHH-----------      403
XP_010693256.1      APAVPWMNPQTGAGYPPNGYSTNAN--MPPQTHAAPAPSWDPSMQPGRR-------TFVS      402

StPTB1              STIRISFSQLQSI*----------------------------------------------      441
StPTB6              SIIRISFSQVQSI-----------------------------------------------      444
XP_010681101.1      SVIRISFSQSQAI-----------------------------------------------      443
XP_010680298.1      SIVRISFSQSQST-----------------------------------------------      445
```

```
XP_010693257.1      ------------------------------------------------------------     314
XP_010671511.1      --MQMPMQNYQYMPPGAAPPGPPGTMPPGSAPPGSMQPGSMHMQNPNGLPQPPAMHPYSQ     450
KMT16075.1          --MQMPMQNYQYMPPGAAPPGPPGTMPPGSAPPGSMQPGSMHMQNPNGLPQPPAMHPYSQ     461
XP_010693256.1      VPSTFPGQTYTASPVPAY------ASAPISPAPGA----SMPMTPPAAS-R-ASQAPYYP     450
```

Carrot PTB1/6 types alignment

```
StPTB1              ---------------------------------------------MSDPSKVVHVRNVGH     15
StPTB6              ---------------------------------------------MTEPSKVIHVRNVGQ     15
XP_017247842.1      ---------------------------------------------MAEPSKVIHVRNVGH     15
XP_017241710.1      ----------------------------------MSNPNQPQFRYTQTPSKVLHLRNLPW     26
XP_017242965.1      ---------------------------------MASVSSQPQFRYTQPPSKVLHLRNLPW     27
XP_017246841.1      ---------------------------------MSSVSSQPQFRYTQPPSKVLHLRNLPW     27
KZM98104.1          MGGQQRIKKYILGPDISLVSTVLCLDFVIVESVMSSVSSQPQFRYTQPPSKVLHLRNLPW     60
KZN02333.1          ----------------------------------MSNPNQPQFRYTQTPSKVLHLRNLPW     26
                                                              ****:*:**:

StPTB1              EISENDLLQLFQPFGVIT--KLVMLRAKNQALLQMQDVPSAVKALQFYSN--VQPSIRGR     71
StPTB6              EISENDLLQLFQPFGVIT--KLVMLRAKNQALLQMQDIAAAVNAMQFYSN--VQPSIRGR     71
XP_017247842.1      EISENDLLQLFQPFGVIT--KLVMLRAKNQALLQMQDVTTAVNALQFYTN--VQPSIRGR     71
XP_017241710.1      ECIEEELVELCKPFGKIVNTKCNVGANRNQAFVEFVELNQAINMVSYYASSSEPASIRGK     86
XP_017242965.1      ECTEDELIELGKPFGKVVNTKCNVGANRNQAFIEFAELNQAIAMISYYASSSEPAQVRGK     87
XP_017246841.1      ECTEEELIELGKPFGKVVNTKCNVGANRNQAFIEFAELNQAIAMISYFASSSEAAQVRGK     87
KZM98104.1          ECTEEELIELGKPFGKVVNTKCNVGANRNQAFIEFAELNQAIAMISYFASSSEAAQVRGK     120
KZN02333.1          ECIEEELVELCKPFGKIVNTKCNVGANRNQAFVEFVELNQAINMVSYYASSSEPASIRGK     86
                    *  *::*::* :*** :.  *  :   :***:::: ::  *:  :.:::.     .:**:

StPTB1              NVYVQFSSHQELTTMDQNAQGRGDEPNRILLVTIHHML-YPITVDVLHQVFSPHGFVEKI     130
StPTB6              SVYVQFSSHQELTTVDQNAQGRGDEPNRILLVSIHHVL-YPITVEVLHQVFSPHGIVEKI     130
XP_017247842.1      NVYIQFSSHQELTTVEQNAQGRGDEPNRILLVTIHHML-YPITVEVLHQVFSPHGFVEKI     130
XP_017241710.1      HVYIQYSNRHEIV----NNKGPGDVPGNVLLVTIEGVEAGDVSIDVIHLVFSAFGFVHKI     142
XP_017242965.1      TVYLQYSNRQEIV----NNKTTADVAGNVLLVTIEGNDARLVSIEVLHVVFSAFGFVHKI     143
XP_017246841.1      TVYLQYSNRQEIV----NNKTTADVAGNVLLVTIEGNDARLVSIEVLHVVFSAFGFVHKI     143
KZM98104.1          TVYLQYSNRQEIV----NNKTTADVAGNVLLVTIEGNDARLVSIEVLHVVFSAFGFVHKI     176
KZN02333.1          HVYIQYSNRHEIV----NNKGPGDVPGNVLLVTIEGVEAGDVSIDVIHLVFSAFGFVHKI     142
                     **:*:*.::*:.    * :  .*  ..:***:*.      ::::*:* *** .*:*.**

StPTB1              VTFQKSAGFQALIQYQVQQSSVSARNSLQGRNIY-------DGCCQLDIQFSNLDELQVN     183
StPTB6              VTFQKSAGFQALIQYELTQTAISARNSLQGRNIY-------DGCCQLDIQFSNLDELQVS     183
XP_017247842.1      VTFQKSAGFQALIQFQLKQSAVTARNSLQGRNIY-------DGCCQLDIQFSNLDELQVN     183
XP_017241710.1      ATFEKAAGFQALIQFTDAETALSAREALDGRSIPRYLLPEHVGSCNLRISYSAHTDLNIK     202
XP_017242965.1      TTFEKTAGFQALVQFTDSETASSAKDALDGRSIPSYLIPE-LAPCSLRITYSAHTDLSVK     202
XP_017246841.1      TTFEKTAGFQALVQFTDSETASSAKDALDGRSIPSYLIPE-LSPCSLKITYSAHTDLSVK     202
KZM98104.1          TTFEKTAGFQALVQFTDSETASSAKDALDGRSIPSYLIPE-LSPCSLKITYSAHTDLSVK     235
KZN02333.1          ATFEKAAGFQALIQFTDAETALSAREALDGRS----------------------------     174
                    .**:*:******:*:   ::: :*:::*:**.

StPTB1              YNNERSRDYTNPNLPSEQKGKSSQQGY---GDMYSFQGSGAHPGGFPQMGNAEAIAAAFA     240
StPTB6              YNNERSRDFTNPNLPSEPKGKSPQQGYGDAGAMYPWQGSGPRGVGFPQMGNAAAIATAFP     243
XP_017247842.1      YNNERSRDFTNPSLPAEQRGRSSQPGYVDAGGVYGFQPSGVRPVGFPQMDNAAAIAAAFG     243
XP_017241710.1      FQSHRSRDYTNPYLPVNSTAIEGFVQP-----VVGPDGKKKE------------------     239
```

```
XP_017242965.1      FQSHRSRDYTNPNLPVNPSAIDATGQI-----SKGLDGKKLE------------------      239
XP_017246841.1      FQSHRSRDYTNPSLPVNPSAIDVTGQI-----SMGLDGKRLE------------------      239
KZM98104.1          FQSHRSRDYTNPSLPVNPSAIDVTGQI-----SMGLDGKRLE------------------      272
KZN02333.1          -----------------------------------PDGKKKE------------------      181
                                                    : .  .

StPTB1              GGLPPGISGINDRCTILVSN--LNSDRINEDKLFNLCSLYGNIVSIKILRN-KPDHALVQ      297
StPTB6              SGLPPGISGTNDRCTIIVSN--VNPDRIDEDKLFNLFSIYGNIVRIKHLRN-KPDHALVQ      300
XP_017247842.1      GGLPPGITGTNDRCTILVSN--LNTDKIDEDKLFNLFSLYGNIVRIKLLRN-KPDHALVQ      300
XP_017241710.1      ----P-------ESNVLLASIENRIYDVTVDVLNTVFSAFGTVQKIAIFEKNATTQALIQ      288
XP_017242965.1      ----P-------ESNVLLASIENMPYEVTLEVLHMVFSAFGTVLKIAMFDKNGGVQALIQ      288
XP_017246841.1      ----P-------ESNVLLASIENMPYELTLDVLHMVFSTFGTVLKIAMFDKNGGIQALVQ      288
KZM98104.1          ----P-------ESNVLLASIENMPYELTLDVLHMVFSTFGTVLKIAMFDKNGGIQALVQ      321
KZN02333.1          ----P-------ESNVLLASIENRIYDVTVDVLNTVFSAFGTVQKIAIFEKNATTQALIQ      230
                        *       ...::::.       :  : *  : * :*.:  *  : :    :**:*

StPTB1              LGDGFQAELAVHFLKGAMLFEK---RLEVNFSKYPNITTGPDTHDYSNSNLNRFNRNAAK      354
StPTB6              MGDGFQAELAVHFLKGAMLFGQ---RLEVNYSKYPNINTGPETRDYSNSNLNRFNRNAAK      357
XP_017247842.1      MGDGFQAELAVHFLKGATLFEK---RLEVNFSKHPNITTGTETHEYSNSNLNRFNRNAAK      357
XP_017241710.1      YPDINIAAAAKDALEGHCIYDGGYCKLHISYSRHTDLNVKAFS-------------DKSR      335
XP_017242965.1      YPDIQTAVVAKEALEGHCIYDGGYCKLHISYSRHTDLSIKVNN-------------DRSR      335
XP_017246841.1      YPDVQTAVVAKQALEGHCVYDGGYCKLHISFSRHTDLSIKVNN-------------NRSR      335
KZM98104.1          YPDVQTAVVAKQALEGHCVYDGGYCKLHISFSRHTDLSIKVNN-------------NRSR      368
KZN02333.1          YPDINIAAAAKDALEGHCIYDGGYCKLHISYSRHTDLNVKAFS-------------DKSR      277
                      *   *  * . *:*  ::     :*.:.:*:: ::.    .             : ::

StPTB1              NYRYCCSPTKMIHLSSLPQDVTEAEIIAHLEEHGPIINSKLFEMNGKQQA-----LVLFD      409
StPTB6              NYRYCCSPTKMIHVSSLHQDVTEEEIVAHLEEHGPIVNTKLFEMNGKKQA-----LILFN      412
XP_017247842.1      NYRYCCSPTKMIHLSTLPQDVTEEEIVTHLEEHGTIANTKVFDMNGKKQA-----LVMFE      412
XP_017241710.1      DYTVPESGF----AAALPAGATVW-QN---PHAAPVFTASEFVGVNYAQPQGPHGHVNYM      387
XP_017242965.1      DYTIPMSSV----LSTQPSILGQL-PPAMVSSGVPQYNGSHYASAHQGHP----------      380
XP_017246841.1      DYTIPNVPL----LSTQPSMLAQQ-SPSLLGPGGPQYNATQFAPVHEGQA----------      380
KZM98104.1          DYTIPNVPL----LSTQPSMLAQQ-SPSLLGPGGPQYNATQFAPVHEGQA----------      413
KZN02333.1          DYTVPESGF----AAALPAGATVW-QN---PHAAPVFTASEFVGVNYAQPQGPHGHVNYM      329
                    :*            ::                     . . :      :

StPTB1              KEEQA--TEALVCKNA----TSLGSSTIRISFSQLQSI*---------------------      441
StPTB6              NEEQA--TEALVCQHA----TSLGGSIIRISFSQVQSI----------------------      444
XP_017247842.1      TEEQA--TEALVCKHA----TSLGGQMIRISFSQLQNI----------------------      444
XP_017241710.1      QPLQGPPGQGPPGQGPPGQGPP--GQG-PPGQGPPGQAPS-WDPAMQLSGPSFVSGSSTL      443
XP_017242965.1      --VHPPSS-GW-SAGPPAVPQPMPGQMHNPQYMPPASMPSEYGHQMMHSPNGFQ------      430
XP_017246841.1      --MPQPPS-GW-NSGAPAGPQPMQGQMHPHHYMP-ANMPSEYGHSVMHNPNSFH------      429
KZM98104.1          --MPQPPS-GW-NSGAPAGPQPMQGQMHPHHYMP-ANMPSEYGHSVMHNPNSFH------      462
KZN02333.1          QPLQGPPGQGPPGQGPPGQGPP--GQG-PPGQGPPGQAPS-WDPAMQLSGPSFVSGSSTL      385
                             .              ..          .

StPTB1              ------------------------------------------------------------      441
StPTB6              ------------------------------------------------------------      444
XP_017247842.1      ------------------------------------------------------------      444
XP_017241710.1      PGQTYGPTSGQVYNPMGSPAGSSPYGPPSGQVYNPMSSPAGSSPLGQKTQVNPSSSASGG      503
XP_017242965.1      ------------------NAGTYPRYPPQ-------------------------------      441
```

```
XP_017246841.1      ------------------HAGPYPHYPPQ-------------------------------      440
KZM98104.1          ------------------HAGPYPHYPPQ-------------------------------      473
KZN02333.1          PGQTYGPTSGQVYNPMGSPAGSSPYGPPSGQVYNPMSSPAGSSPLGQKTQVNPSSSASGG      445


StPTB1              ------------------------    441
StPTB6              ------------------------    444
XP_017247842.1      ------------------------    444
XP_017241710.1      QPGNPSNMQHGSSSPRNHHPPYYR    527
XP_017242965.1      ------------------------    441
XP_017246841.1      ------------------------    440
KZM98104.1          ------------------------    473
KZN02333.1          QPGNPSNMQHGSSSPRNHHPPYYR    469
```

Radish PTB1/6 types alignment

```
StPTB1              ------------MSDPSKVVHVRNVGHEISENDLLQLFQPFGVIT--KLVMLRAKNQALL      46
StPTB6              ------------MTEPSKVIHVRNVGQEISENDLLQLFQPFGVIT--KLVMLRAKNQALL      46
XP_018451916.1      ------------MAESSKVIHVRNVGHEISENDLLQLFQPFGVIT--KLVMLRAKNQALL      46
XP_018441449.1      ---MSSSSQFRYTQTPSKVVHLRNLPWECVEEELIDLCKRFGKIVNTKTNVGANRNQAFV      57
XP_018441450.1      ---MSSSSQFRYTQTPSKVVHLRNLPWECVEEELIDLCKRFGKIVNTKTNVGANRNQAFV      57
XP_018472559.1      MSSVSSQQQFRYTQTPSKVLHLRNLPWECTEEELIELGKPFGTVVNTKCNVGANKNQAFI      60
XP_018488837.1      ----MSSSQFRYTQTPSKVVHLRNLPWECVEEELIDLCKRFGKIVNTKTNVGANRNQAFV      56
XP_018488838.1      ----MSSSQFRYTQTPSKVVHLRNLPWECVEEELIDLCKRFGKIVNTKTNVGANRNQAFV      56
XP_018488836.1      ----MSSSQFRYTQTPSKVVHLRNLPWECVEEELIDLCKRFGKIVNTKTNVGANRNQAFV      56
XP_018463656.1      ----MSSSQFRYTQTPSKVVHLRNLPWECVEEELIDLCKRFGKIVNTKTNVGANRNQAFV      56
                                    ***:*:**:  *  *::*::* : ** :.  *  :   :***::

StPTB1              QMQDVPSAVKALQFYSNV--QPSIRGRNVYVQFSSHQELTTMDQNAQGRGDEPNRILLVT      104
StPTB6              QMQDIAAAVNAMQFYSNV--QPSIRGRSVYVQFSSHQELTTVDQNAQGRGDEPNRILLVS      104
XP_018451916.1      QMQDVSSAITALQFFTTV--QPTIRGRNVYIQFSSHQELTTAEQNIHGREDEPNRILLVT      104
XP_018441449.1      EFAEVNQAISMVSYYASSSEPAQIRGKTVYIQYSNRHEIVNN----QSPGEVPGNVLLVT      113
XP_018441450.1      EFAEVNQAISMVSYYASSSEPAQIRGKTVYIQYSNRHEIVNN----QSPGEVPGNVLLVT      113
XP_018472559.1      EFEDLNQAIQMISFYASSSEPAQVRGKTVYLQYSNRQEIVNN----KTAADVVGNVLLVT      116
XP_018488837.1      EFAELNQAISMVSYYASSSEPAQIRGKTIYIQYSNRHEIVNN----QSPGEVPGNVLLVT      112
XP_018488838.1      EFAELNQAISMVSYYASSSEPAQIRGKTIYIQYSNRHEIVNN----QSPGEVPGNVLLVT      112
XP_018488836.1      EFAELNQAISMVSYYASSSEPAQIRGKTIYIQYSNRHEIVNN----QSPGEVPGNVLLVT      112
XP_018463656.1      EFAELNQAISMVSYYASSSEPAQIRGKTIYIQYSNRHEIVNN----QSPGEVPGNVLLVT      112
                    :: ::  *:  :.:::.      :**:.:*:*:*.::*:..     :   :  ..:***:

StPTB1              IHHM-LYPITVDVLHQVFSPHGFVEKIVTFQKSAGFQALIQYQVQQSSVSARNSLQGRNI      163
StPTB6              IHHV-LYPITVEVLHQVFSPHGIVEKIVTFQKSAGFQALIQYELTQTAISARNSLQGRNI      163
XP_018451916.1      VHHM-LYPITVDVLHQVFSPYGFVEKIVTFQKSAGFQALIQYQAQQCAASARTSLQGRNI      163
XP_018441449.1      FEGVESHHVSIDVIHLVFSAYGFVHKIATFEKAAGFQALVQFTDVDTALAARTALDGRSI      173
XP_018441450.1      FEGVESHHVSIDVIHLVFSAYGFVHKIATFEKAAGFQALVQFTDVDTALAARTALDGRSI      173
XP_018472559.1      VEGEDARMVSIDVLHLVFSAFGFVHKITTFEKTAGYQALVQFTDAETATSARTSLDGRNI      176
XP_018488837.1      FEGVESHHVSIDVIHLVFSAFGFVHKIATFEKAAGFQALVQFTDVETASAARSALDGRSI      172
XP_018488838.1      FEGVESHHVSIDVIHLVFSAFGFVHKIATFEKAAGFQALVQFTDVETASAARSALDGRSI      172
XP_018488836.1      FEGVESHHVSIDVIHLVFSAFGFVHKIATFEKAAGFQALVQFTDVETASAARSALDGRSI      172
XP_018463656.1      FEGVESHHLSIDVIHLVFSAFGFVHKIATFEKAAGFQALVQFTDVETASAARSALDGRSI      172
                    ..      ::::*:* *** .*:*.**.**:*:**:***:*:   : : :**.:*:**.*
```

```
StPTB1          YD-------GCCQLDIQFSNLDELQVNYNNERSRDYTNPNLPSEQKGKSSQ--QGYGD--      212
StPTB6          YD-------GCCQLDIQFSNLDELQVSYNNERSRDFTNPNLPSEPKGKSPQ--QGYGDAG      214
XP_018451916.1  YD-------GCCQLDIQFSNLEELQVNYNNDRSRDYTNPNLPSEQKGRLPH--PGYGDAG      214
XP_018441449.1  PKYLLPEHVGSCNLRMSYSAHTDLNIKFQSHRSRDYTDPYLPVNQTAMDGSMQPALGADG      233
XP_018441450.1  PKYLLPEHVGSCNLRMSYSAHTDLNIKFQSHRSRDYTDPYLPVNQTAMDGSMQPALGADG      233
XP_018472559.1  PSYLLPEEVSPCSLKITYSAHTDLTVKFQSHRSRDYTNPYLPVAPSAIDSTGQVVVGVDG      236
XP_018488837.1  PKYLLPEHVASCNLRMSYSAHTDLNIKFQSHRSRDYTNPYLPVNHTAMDGSMQPALGADG      232
XP_018488838.1  PKYLLPEHVASCNLRMSYSAHTDLNIKFQSHRSRDYTNPYLPVNHTAMDGSMQPALGADG      232
XP_018488836.1  PKYLLPEHVASCNLRMSYSAHTDLNIKFQSHRSRDYTNPYLPVNHTAMDGSMQPALGADG      232
XP_018463656.1  PKYLLPEHVASCNLRMSYSAHTDLNIKFQSHRSRDYTNPYLPVNHTAMDGSMQPSLGADG      232
                 .       . *.* : :*   :* :.::..****:*:* **   ..         *

StPTB1          -MYSFQGSGAHPGGFPQMGNAEAIAAAFAGGLPPGISGINDRCTILVS--NLNSDRINED      269
StPTB6          AMYPWQGSGPRGVGFPQMGNAAAIATAFPSGLPPGISGTNDRCTIIVS--NVNPDRIDED      272
XP_018451916.1  V------------AYPQMANTSAIAAAFGGGLPPGITGTNDRCTILVS--NLNTDSVDED      260
XP_018441449.1  KR---------------VET---------------------QSNVLLALIENMQYAVTVD      257
XP_018441450.1  KR---------------VET---------------------QSNVLLALIENMQYAVTVD      257
XP_018472559.1  RK---------------MEP---------------------ESNVLLASIENMQYAVTLD      260
XP_018488837.1  KK---------------VET---------------------QSNVLLALIENMQYAVTVD      256
XP_018488838.1  KK---------------VET---------------------QSNVLLALIENMQYAVTVD      256
XP_018488836.1  KK---------------VET---------------------QSNVLLALIENMQYAVTVD      256
XP_018463656.1  KK---------------VET---------------------QSNVLLALIENMQYAVTVD      256
                                 :                       ...::::  :     :  *

StPTB1          KLFNLCSLYGNIVSIKILR-NKPDHALVQLGDGFQAELAVHFLKGAMLFEK---RLEVNF      325
StPTB6          KLFNLFSIYGNIVRIKHLR-NKPDHALVQMGDGFQAELAVHFLKGAMLFGQ---RLEVNY      328
XP_018451916.1  KLFNLFSLYGNIVRIKLLR-NKPDHALVQMGDGFQAELAVHFLKGAMLFGK---RLEVNY      316
XP_018441449.1  VLHTVFSAYGTVQKIAIFEKNGSTQALIQYSDIPTATIAKEALEGHCIYDGGYCKLRLTY      317
XP_018441450.1  VLHTVFSAYGTVQKIAIFEKNGSTQALIQYSDIPTATIAKEALEGHCIYDGGYCI-----      312
XP_018472559.1  VLHTVFVAFGAVQKIAMFDKNGGVQALIQYPDVQTAVVAKGALEGHCIYEGGFCKLHITY      320
XP_018488837.1  VLHTVFSAYGTVQKIAIFEKNGSTQALIQYSDIATAAIAKEALEGHCIYDGGYCKLRLSY      316
XP_018488838.1  VLHTVFSAYGTVQKIAIFEKNGSTQALIQYSDIATAAIAKEALEGHCIYDGGYCI-----      311
XP_018488836.1  VLHTVFSAYGTVQKIAIFEKNGSTQALIQYSDIATAAIAKEALEGHCIYDGGYCKLRLSY      316
XP_018463656.1  VLHTVFSAYGTVQKIAIFEKNGSTQALIQYSDIATAAIAKEALEGHCIYDGGYCKLRLSY      316
                 *..:   :* :  *  :  *   :**:*  *   * :*   *:*  ::

StPTB1          SKYPNITTGP---DTHDYSNSNLNRFNRNAA-KNYRYCCSPTKMIHLSSLPQDV-TEAEI      380
StPTB6          SKYPNINTGP---ETRDYSNSNLNRFNRNAA-KNYRYCCSPTKMIHVSSLHQDV-TEEEI      383
XP_018451916.1  SKHPNITPGT---DSHDYVNSNLNRFNRNAA-KNYRYCCSPTKMIHLSTLPQDV-TEEEV      371
XP_018441449.1  SRHTDLNVKAFSDKSRDYTLPDLSQLVGQKVPGVAA------------------------      353
XP_018441450.1  ------------------------------------------------------------      312
XP_018472559.1  SRHTDLSIKVNNDRSRDYTMPDPAVAMAPQP-GHNPYPS---------NSPQYQGADASH      370
XP_018488837.1  SRHTDLNVKVHKIGCFLE------------------------------------------      334
XP_018488838.1  ------------------------------------------------------------      311
XP_018488836.1  SRHTDLNVKAFSDKSRDYTLPDLSLLVGQKVPGVAE------------------------      352
XP_018463656.1  SRHTDLNVKAFSDKSRDYTLPDLSLLVGQKVPGVAE------------------------      352

StPTB1          IAHLEEHGPIINSKLFEMNGKQQALVLFDKEEQATEALVCKNATS----LGSSTIRISFS      436
StPTB6          VAHLEEHGPIVNTKLFEMNGKKQALILFNNEEQATEALVCQHATS----LGGSIIRISFS      439
XP_018451916.1  VNHVQEHGAILNTKVFEMNGKKQALVQFENEEEAAEALVCKHATS----LGGSIIRISFS      427
```

```
XP_018441449.1        -ASGPT-DGWH-----------NGQVQTQYA---GSSYMYSPADPTGASPSSGH-PPYYG      396
XP_018441450.1        ------------------------------------------------------------      312
XP_018472559.1        YQQQQPQGGWG-----------Q-----QSGGQGHNPYMGGPPSMHQGGPGGYMPPHHYG      414
XP_018488837.1        --------LWE-----------RD------------------------------------      339
XP_018488838.1        ------------------------------------------------------------      311
XP_018488836.1        -ASAPTTGGWQ-----------NGQVQTQYAGYGGSSYMYPSADPTGASPSSGH-PPYYG      399
XP_018463656.1        -ASAPTTGGWQ-----------NGQVQTQYAGYGGSPYMYPSADPTGASPSSGH-PPYYG      399

StPTB1                QLQS--I* 441
StPTB6                QVQS--I- 444
XP_018451916.1        QLQT--I- 432
XP_018441449.1        -------- 396
XP_018441450.1        -------- 312
XP_018472559.1        SGPSGPMH 422
XP_018488837.1        -------- 339
XP_018488838.1        -------- 311
XP_018488836.1        -------- 399
XP_018463656.1        -------- 399
```

Sweet potato PTB1/6 types alignment

```
StPTB1                ------------MSDPSKVVHVRNVGHEISENDLLQLFQPFGVITK--LVMLRAKNQALL      46
StPTB6                ------------MTEPSKVIHVRNVGQEISENDLLQLFQPFGVITK--LVMLRAKNQALL      46
itf09g10450.t1        ------------MTEPSKVIHVRNVGHEISENDLLQLFQPFGVITK--LVMLRAKNQALL      46
itf05g19430.t1        MSSVSSQPQFRYTQPPSKVLHLRNLPWECTEEELIELGKPFGRVVNTKCNVGANRNQAFI      60
itf05g16040.t1        -MSTSGQPQFRYTQTPSKVLHLRNLPWECIEEELVELCKPFGRIVNSKCNVGSNRNQAFV      59
itf12g25960.t1        MSSVSSQPQFRYTQPPSKVLHLRNLPWECSEEELIELGKPFGKVVNTKCNVGANRNQAFI      60
itf05g19430.t2        MSSVSSQPQFRYTQPPSKVLHLRNLPWECTEEELIELGKPFGRVVNTKCNVGANRNQAFI      60
                                     ****:*:**:  *  *::*::* :*** :.:    :   :***::

StPTB1                QMQDVPSAVKALQFYSNV--QPSIRGRNVYVQFSSHQELTTMDQNAQGRGDEPNRILLVT      104
StPTB6                QMQDIAAAVNAMQFYSNV--QPSIRGRSVYVQFSSHQELTTVDQNAQGRGDEPNRILLVS      104
itf09g10450.t1        QMQDVPSAVNALQFYTNV--QPSIRGRNVYIQFSSHQELTTMDQNTQSRGDEPNRILLVT      104
itf05g19430.t1        EFAEQNQAIAMISYYASSSEPAQVRGKTVYLQYSNRQEIVN----NKTTADVAGNVLLVT      116
itf05g16040.t1        EFADLNQAINMATYYASSSEPAQIRGKTVYIQYSNRHEIVN----NKSPGDIPGNVLLVT      115
itf12g25960.t1        EFAELNQAIAMISYYASSSEPAQVRGKTVYLQYSNRQEIVN----NKTTADVAGNVLLVT      116
itf05g19430.t2        EFAEQNQAIAMISYYASSSEPAQVRGKTVYLQYSNRQEIVN----NKTTADVAGNVLLVT      116
                      :: :   *:    :*:.     .:**:.**:*:*.::*:..     :  .*  ..:***:

StPTB1                IHHML-YPITVDVLHQVFSPHGFVEKIVTFQKSAGFQALIQYQVQQSSVSARNSLQGRNI      163
StPTB6                IHHVL-YPITVEVLHQVFSPHGIVEKIVTFQKSAGFQALIQYELTQTAISARNSLQGRNI      163
itf09g10450.t1        IHHML-YPITVEVLHQVFSPHGFVEKIVTFQKSAGFQALIQYQANQSAVSARNALQGRNI      163
itf05g19430.t1        IEGNDARLVSIDILHLVFSAFGFVHKITTFEKTAGFQALVQFSDAETATSAKDALDGRSI      176
itf05g16040.t1        IEGVEAGDVSIDVIHLVFSAFGFVHKIATFEKAAGFQALVQFTDIETAATAREALDGRSI      175
itf12g25960.t1        IEGNDARLVSIDVLHLVFSAFGFVHKITTFEKTAGFQALVQFSDAVTATSAKDALDGRSI      176
itf05g19430.t2        IEGNDARLVSIDILHLVFSAFGFVHKITTFEKTAGFQALVQFSDAETATSAKDALDGRSI      176
                      *.      ::::::* *** .*:*.**.**:*:******:*:    :: :*:::*:**.*

StPTB1                Y-------DGCCQLDIQFSNLDELQVNYNNERSRDYTNPNLPSEQKGKSSQQGYGDMYSF      216
StPTB6                Y-------DGCCQLDIQFSNLDELQVSYNNERSRDFTNPNLPSEPKGKSPQQGYGDAGAM      216
```

```
itf09g10450.t1      Y-------DGCCQLDIQFSNLDELQVNYNNDRSRDFTNPNLPSEQKGKSSQHGYGDAGGM      216
itf05g19430.t1      PSYLIPD-LGPCSLKITYSAHTDLSVKFQSHRSRDYTNPHLPVAPSAIDASGQ-------      228
itf05g16040.t1      PRYLLPQHVSNCHLRISFSAHTDLNIKFQSNRSRDYTNPYLPVNPTAMEGFVQ-------      228
itf12g25960.t1      PSYLIPE-LGPCSLRITYSAHTDLTVKFQSHRSRDYTNPLLPVAPSAIDANSQ-------      228
itf05g19430.t2      PSYLIPD-LGPCSLKITYSAHTDLSV-------KDYTNPHLPVAPSAIDASGQ-------      221
                             . * * * :*   :* :       :*:*** **   .. .

StPTB1              ---QGSGAHPGGFPQMGNAEAIAAAFAGGLPPGISGINDRCTILVSNL--NSDRINEDKL      271
StPTB6              YPWQGSGPRGVGFPQMGNAAAIATAFPSGLPPGISGTNDRCTIIVSNV--NPDRIDEDKL      274
itf09g10450.t1      ---YS-LQAPVGFPQMGNAAAIAAAFPGGLPPGISGTNDRCTILVSNL--NPDRIDEDKL      270
itf05g19430.t1      --------FTVGLDGKK------------LDP-------ESNVLLAAIENMQYAVTVDVL      261
itf05g16040.t1      --------PVVGADGKK------------KEP-------ESNVLLASIENMQYAVTVDVL      261
itf12g25960.t1      --------FSVGLDGKK------------LEP-------ESNVLLASIENMQYAVTLDVL      261
itf05g19430.t2      --------FTVGLDGKK------------LDP-------ESNVLLAAIENMQYAVTVDVL      254
                               *                   *       ...:::: :      :  * *

StPTB1              FNLCSLYGNIVSIKILRN-KPDHALVQLGDGFQAELAVHFLKGAMLFEK---RLEVNFSK      327
StPTB6              FNLFSIYGNIVRIKHLRN-KPDHALVQMGDGFQAELAVHFLKGAMLFGQ---RLEVNYSK      330
itf09g10450.t1      FNLFSIYGNIVRIKLLRN-KPDHALVQMGDGFQAELAVHFLKGGMLFGK---KLEVNFSK      326
itf05g19430.t1      HTVFSAFGPVLKIAMFDKNGGLQALVQYPDVQTAVAAKDALEGHCIYEGGFCKLHITYSR      321
itf05g16040.t1      HTVFSAFGTVQKIAIFEKNGGTQAFIQYPDVTTATVAKDALEGHCIYDGGYCKLHLSYSR      321
itf12g25960.t1      HTVFAAFGPVLKIAMFDKNGGLQALIQYPDVQTAVVAKEALEGHSIYEGGYCKLHITYSR      321
itf05g19430.t2      HTVFSAFGPVLKIAMFDKNGGLQALVQYPDVQTAVAAKDALEGHCIYEGGFCKLHITYSR      314
                    ..: : :* :  *  : :    :*::*  *   *  * . *:*  ::     :*.:.:*:

StPTB1              YPNITTGPDTHDYSNSNLNRFNRNAAKNYRYCCSPTKMIHLSSLPQDVTEAE---IIAHL      384
StPTB6              YPNINTGPETRDYSNSNLNRFNRNAAKNYRYCCSPTKMIHVSSLHQDVTEEE---IVAHL      387
itf09g10450.t1      HPNITTGPDTHDYSNSNLNRFNRNAAKNYRYCCSPTKMIHLSTLPQDVSETE---IVAHL      383
itf05g19430.t1      HTDLSIKVN-------------NDRGRDYTIPNAPMSSQPPVLLGQQQSP--TGGPGAHP      366
itf05g16040.t1      HTDLNVKAY-------------SDKSRDYRVPESGLPPLQQASALPNTAPA--------W      360
itf12g25960.t1      HTDLSIKVN-------------NDRSRDYTIPNAPMLNAQPSNMGGQQPPPSVGGPGGHP      368
itf05g19430.t2      HTDLSIKVN-------------NDRGRDYTIPNAPMSSQPPVLLGQQQSP--TGGPGAHP      359
                    : ::.                  : .::*    :            :

StPTB1              EEH-------------GPIINSKLFEMNGKQQALVL----FDKEEQATEAL---VCKNAT      424
StPTB6              EEH-------------GPIVNTKLFEMNGKKQALIL----FNNEEQATEAL---VCQHAT      427
itf09g10450.t1      EEH-------------GTIVNSKLFEMNGKKQALVL----FENEEQATEAL---VCKNAS      423
itf05g19430.t1      YNATQYASAPNVHAA-PQAASSWNFVGAAGPPPMSMQMHN-----------PPYMGPANV      414
itf05g16040.t1      QNHPQYVSVHSAPPATMPMQNPWDPTMQGGRPAFISAPTTFPGQEYASPPVPAYTTAAPI      420
itf12g25960.t1      YNGNQYAPAHDGHAA-PQPSASWNSGNVGGPPPMGMQMHN-----------SPYMPSASG      416
itf05g19430.t2      YNATQYASAPNVHAA-PQAASSWNFVGAAGPPPMSMQMHN-----------PPYMGPANV      407
                     :                          .    :

StPTB1              SLGSST---IRISFSQLQSI*-----------      441
StPTB6              SLGGSI---IRISFSQVQSI------------      444
itf09g10450.t1      SLGGST---IRISFSQLQNI*-----------      440
itf05g19430.t1      HN-----------------QNRPAMPPYQLR*      428
itf05g16040.t1      PPGSSPTSQPNAPFGASHPAHQGIPPPGQPR*      451
itf12g25960.t1      PQG----------YNQSGLPHSGAMPPYQPR*      437
itf05g19430.t2      HN-----------------QNRPAMPPYQLR*      421
```
