# Supplementary material for: Conservation of polypyrimidine tract binding proteins and their putative target RNAs in several storage root crops
Source: BMC Genomics. 2018 Feb 7;19:124. doi: 10.1186/s12864-018-4502-7 (PMC5803842; doi:10.1186/s12864-018-4502-7)
Supplement: Supplementary file 5 — Cytosine/uracil motifs in the 3´ UTRs of BEL5- and POTH1-like mRNAs. (PDF 148 kb) [file 12864_2018_4502_MOESM5_ESM.pdf]

Additional file: Table S3. Cytosine/uracil (CU) motifs present in 3´ UTRs of potato mobile RNAs, *StBEL5* (A) and *POTH1* (B), and in orthologues of the storage root crops, sweet potato, cassava, carrot, radish and sugar beet. Only CU motifs of three nucleotides or more are underlined and highlighted in yellow. Gene IDs for *StBEL5* and *POTH1* orthologues were included along with potato *POTH1* and *StBEL5*. The 3' UTRs of *StBEL11* and *StBEL29* were included as confirmed mobile RNAs (C). As a negative control, the 3´ UTRs of two non-mobile *StBEL* mRNAs, *StBEL14* and *-22*, were also compared. Both contain only two CU motifs (D). All these accession nos. are also represented in Tables 2 and 3.

(A) The presence of cytosine/uracil (CU) motifs present in the 3´ UTR of *BEL5-like* mRNAs from a range of storage root crops.

***StBEL5* in potato (PGSC0003DMG400005930) 3´ UTR= 501bp No. of motifs= 17**
AUACCGAAAGUCUCGUAUUGAUAGCUGAAAAGAUAAAAGGAAGUUAGGGAUUACUCUU
AUAUUGUGUGAGGCCUUCUGGCCCAAGUCGGAGGACCCAAUUUGAUACAACCUAUCAUA
GGAGAAAAGAAGUGGAGACUAAAUUAAAGUAACAAAAUUUUAAAGCACACUUUCUAGU
AUAUAUACUUCUUUUUUUUAUAGUAUAGAAAAGAAGAGAUUUUGUGCUUUAGUGUAUA
GAUAGAGUCUACUUAGUAUUAGGUUAUACUUCUAGUUCCUUGAGAAGAUUGAUACAAC
UAGUAGUAUUUUUUUUCUUUUGGGUUGGCUUGGAGUACUAUUUUAAGUUAUUGGAAAC
UAGCUAUAGUAAAUGUUGUAAAGUUGUGUUCCUCUCAAUUUGCAUAUAAUUUGAAAUA
UUUUGUACCUACUAGCUAGUCUCUAAAUUAUGUUUCCAUUGCUUGUAAUUGCAAUUUU
AUUUGAAUUUUGUGCUAUCAUUAUUAGAUUAGCAAA

***ItBEL5-like* in *I. trifida* (itf04g32320.t1) 3´ UTR length= 428 bp No. of motifs: 15**
AAAAAAGGGGUUUAAUUUGCAGGUAAUUUAAUAUAUAUGCAUAUAUGCAUGGAAUCAA
AGAAAUGGACAGAAAAGUAGUAUAUAAUUUUCAUUAAUUAUUACGAACAUCUUCCAUA
AAAUCUUGUACCUAUAUAUACAUAUAUAGCUCCUCCUUUUAGUGCCUAACCUGCAGGAA
AAAAAGAGGAGAUCAGAUGAUUUUAUUAUGGUUAGUGUAAGAUAGGUUUAAGUGAUUG
GUAUAGGUUACACUUUGCAGUGGAACAAAUAUACUACGUAAGUAGAUAUAUAUAAUUA
UAUAUAUCUUCAAAUAUAUAGAUUGGGUGGGUAUUUACGGUUCAUCAACUAUAAUAUA
UCUUUAUGCAAACAUUUGUCAAUGAAUUAAUGAAAACUUUGUAUGCUGUAAAUUUAUU
AUAUAUUCAUCUUAAUUCAAU

***MeBEL5-like* in cassava (Manes.09G045600.1) 3´ UTR length= 333bp No. of motifs: 11**
UGACAAAGCCAAUGCCAUGGAGAAGUCACCAGUGGAGGUCUCCACGGCUCCAAGAUCUC
UUCACACUAUUUCUUGCUGUUCCAACAAUUAAUAGGGAACAAGGAUAAAUGCAUGGUA
GUGUAAAGAAAGAACAAAUUUUACAUAGUAUAGGUCUAUACUUUGGCAUAUAACCAUU
UGAGUUCUUAGAUAGCUUAGGAUUAUAUGGGUUGAGGAUUGUAUAUUAUUUUUGCAAG

UUUUUGAAGAUGGAUAGAUGGAUUGAUGUUAUAAGAAUUAGUUAUUGGGGUAUACUAA
UAUAUAUUGAUUGUGAGCAAAUAUGGUGUCAUUCCAUGCUUG

***DcBEL5-like* in carrot (Gene ID: 108227378) 3´ UTR length= 378bp No. of motifs: 12**
AAAGGGGAGUAAGAUCUACACUUUCACUACUUUCUUCAAGUACCUUCAUUAGGAAGAA
GGGAAAGUGAAAAGAGUAGUGUAAAGAAAGGACAUGAAACUUUUUACAUAGACUACAG
GUUUAUAUAUAUACUUGGCACAGAACCGUUUGAGUUCUUAUAGAUAGCCUAGCUAGAU
UAGAACAUACUAUUGUACGGGGAUUGUAUAUUAUUUUGUUUUUUGCAGAUGGGUAGA
UGGAUUGGUAUAAGGAUAAUGUAAACAUAGGUAUUUUGUGGGGGUGGUAUAGAAUUUU
UAUAUUGCUUAAAUGUGUACUUAUGCAGAUUCCAUCUUAUGAAUGGAACAGAAGCACU
AUUAUUUUUAUUGGUAUCAUAUGUUUUGUA

***RsBEL5-like* in radish (Gene ID: 108854534) 3´ UTR length= 316bp No. of motifs: 10**
ACCAUGAGCAACCAACAAAGGUUAUUUAUACAUUAGUACUCAAAUUAGUAUAUAGUUU
UUCUUAUACCAUUGAACCAAAACAAAGAACAAAAUUUAAUUUUAGUCUUUGGUUAUAU
AUAUGGCCGACGCUUGUAUAUCGUUUUUUGUAAUUUGUUUUUUUUUUCGAUUUUUAU
UGGGGGAUGGAUAGAGUUAUAUGUAUAUGGGAUUAGUUACGUGUAAUAGCUUGCUUUU
GGGUGUAUACCAACAUAUAUUUUUACUUUUUUUGUUGUAAUAAUAAGAUAGUAGUCUG
AGUUAUCUAAUAUGCUAUGAUGAUUA

***BvBEL5-like* in sugar beet (Gene ID: 104899855) 3´ UTR length= 450bp No. of motifs: 14**
CAAUAAUACUUUAAAAGGAAGAUUCAAGCAUGAUUAUUAUUGAUUAAGAUAGCCAUCA
ACAUGAUAAAUCUUUAGUUUUUCUUAAUGAACAAGAAUUAAGAUCAUUGAAAAGAAAA
AAACAAAGGUUAACCAAGGGGAUUUGGUAUAGGCUUAUACUUAGGGAUUCAAAUAUAU
UAUUGCUACGUACGUAGCUAAAAAAUGUAGGGGAAGUAGGGAUUGUUGAGAUGUUAAU
GCAUAUAGUAUGUGCAAUUGAAGACUUGAAGUAGUUGGAUAUUGUUGAUUAUAUAUGU
UGUAAGGUAUAGACACAGUCAUGAACUUGAAUUUGUAUAUAUGGAUUGUAAUAGUAUA
UAGAUUUCGUGAAGUUAAAUUAAUCUUUUGAAAUCUAUGUAUAUGUAGUAUGAACUUA
UUAUAACUGCUAAUGCAGAAAAUAUCACUUCUGAUGGUUAUCAA

(B) The presence of cytosine/uracil (CU) motifs present in the 3´ UTR of *POTH1-like* mRNAs from a range of storage root crops.

***POTH1* in potato (PGSC0003DMG400013493) 3´ UTR= 211bp No. of motifs= 7**
GUUUGAAUGGAAAUUGUGAAAAUACUGCUCUUCAUUUCUCUUUUUAUUAUAUAUAAUA
UAUAAAUAGUAUAUUUUUGGGAAAGAAAGAAGUUAUUUUAUUAAUCAAAAUCUCUAUA
AAUAAUGGUAGAGAUUAAUUAAUGUUGAAUUCUUCUUGAUCAUGUAAAUAUUCAAUCU
AGCUAAUUGUCAAAAUUAAUGCUUACCUAAAAAAAAA

***ItPOTH1-like* in *I. trifida* (itf15g13570.t2) 3´ UTR length= 140 bp No. of motifs: 4**
AAUAGGUGAUAGCUAGCUAGUGUCAUGCAUGCUAGUAUGCUCAAAAUCAAAUUGUGUC
CAUAUAAAGAUUCUGAUGUUUAUUGCAGCUAGCUGUUUAAAUCAUAUGGAGUAACAUU
AUAUUCUAUGAGAGUUAUUUUUUU

***MePOTH1-like* in cassava (Manes.12G025600.1) 3´ UTR length= 397bp No. of motifs: 20**
GUUUCAUAUGCAUUCUGGAACCCUGAUAAUCUGUGUUGGCUUCCUGUAUAUUCUGCAAU
GCAAACUCUAGGGGCAUGGUUAUUUCCCCAAGAAGAAAAAGGAAGGGGUGGAGGGGAG
AAUUAGUUGUAUUUUUCGCCAUGUAUAUUCACAUUGAUAGACAUUGAUAAUGAGACGU
UUACAUAGCUUGUAUGAAUAAUUUACCCGUGCUUAUUGUCUGAAUUACUCCUUUUUCU

GUAUGUAUUUAUGUAGUUUUCUGGGGAAAUUUGGUACAUUUGAUCGACUUUGUCGCAG
AUGUUCAAAGAACUGCAGAAUGACAAGGAAAUUGUAAGGAAAAAAGAAUGAUAAUCGA
CUUGAUUUCUCCUCAGAUUGGUGUGAAUUUCAACUGGUGAAGUAUUUA

***DcPOTH1-like* in carrot (Gene ID: 108197298) 3´ UTR length= 253bp No. of motifs: 13**
ACGAAACCAGGUAGAACGCAGAUUCUCGAAAAUAUUCGGAUAUUUGAGCAUGGUUUGG
UUCGGUUCGGUUCGAUUUUUUUUCAUGUUUGUAUAUUCUUAAUAUUACUUGCUUUAAA
CAAGUUGUCAUCUUCUUUUAUGAAUAUAAGAGAAGAUUAACAGUAGUUUCUUAACAGA
UAUGGAACAUGUAUGUUCAGGCACAUGCAACUAGCAAGACUGGAACAUAAUUUUAUUC
UAAAUAUUUAUUAAGAUUAAA

***RsPOTH1-like* in radish (Gene ID: 108814646) 3´ UTR length= 97bp No. of motifs: 3**
AAAUUACGGUAUUUAACGUUGGACUAAAAAGAAAAGAAAUGGGGGUGUUAUUGACAUG
UUUCCUUCGUAUAAAUUCUACUUUGCUAUGCCGACAAAA

***POTH1-like* in sugar beet (Gene ID: 104908392) 3´ UTR length= 248bp No. of motifs: 14**
CUAAGGAUAAGCGAAAGAAAUGAGACAUCUACAAACUCAACCCUUCACAACACACAAGA
AUCAUAUUUAUUCUGGCUGCUUAUCUAUGUAUAUGCUAAGCUAAUGGCAAUCUAGCAA
UAUGAAAAACACUCUUUAGGCUCAUCGGAUGAAAGGAAGGGAAAACAUGCUCCUUCCAC
CAUUUGCAUUCUACUCGAUGCAAACGAUGUACUACUAAUGUAUCUGCAUAAAUGAAAU
GGCUAAUUUCAUAA

(C) 3´ UTRs of other confirmed potato mobile RNAs highlighting the CU motifs.

***StBEL11* in potato (PGSC0003DMG400019635) 3´ UTR= 288bp No. of motifs= 7**
UGAUAUAUAUAAUUUGCAGGUAAAUCAGCUUGAAAUUACAUCAUGACAGGUCUUGAAU
AAAAGAAGGGGAGUUGAGAUUUAGUGAUCAUAUAAAUAUGUAUAGGUAGAAAUUUUAG
UUAGUAUAUAUAGGUUAUACUUCUAGUUUCUUAUGAAGAUACAAGUUUUGUUGUUAUU
UUUGUAUUGAGGUAACUAGCUAGCUUGGAUUAUUUAAAGUUGUGCAUGCAACUAAAGA
AGAAGAAAAAAUAAUCUAUAUAUGCAAACUACAGUAUAUUGUAAAUUUUGUGCUUC

***StBEL29* in potato (PGSC0003DMG400021323) 3´ UTR= 329bp No. of motifs= 11**
UCACAAAAACAAAAACAGGUUUUGGCAACAGACAAACUUCUGUCGCUAAACAAGGACAU
GAUUUAGCGACAGAUAACUUCAGUCGCUAACUUAGCGACUGAAAACUUCUGUCGCUAAG
CAUGAACAUGUAUUAGCGACAUACAGUAUGCAACUGUAUGUCACUAAACAAGAACAUG
AUGAAUUAGUGACGGACAACUUCUGUCGCUAAACAACAAAAAAAAAUCCAUGUUUUAG
UAUAUUGUUUCUCAUUCUAUACAUUGGAGAUGAAGAACCAUUUAAGUUCUUCAAAAUA
GAUAGAUUUUCUAGGUUACUUCUAGAAGAUAUAUAUA

(D) 3´ UTRs of the non-mobile *StBEL* RNAs, *StBEL14* and *-22*, highlighting the CU motifs.

***StBEL14* in potato (PGSC0003DMG400012329) 3´ UTR= 76 bp, No. of motifs= 2**
UACAUUUGGGAAUUUUUUAGGUAUUUUGGGUGAUGACAUGAAUUUUGUUUCUAUAGUU
GGUAAUUCUUGGAUGCUA

***StBEL22* in potato (PGSC0003DMG400022011) 3´ UTR= 74 bp, No. of motifs= 2**
AUUCAAACGCUGCUGCAUAAAUUAUGAUUAUAUAUAUAUUGUCAUCUGAUUAGUUUAC
CGGAUUUUGAUUGCAC
